# Degradation and Transmission of Tau by Autophagic-Endolysosomal Networks and Potential Therapeutic Targets for Tauopathy

**DOI:** 10.3389/fnmol.2020.586731

**Published:** 2020-10-16

**Authors:** Shanya Jiang, Kiran Bhaskar

**Affiliations:** Department of Molecular Genetics and Microbiology, The University of New Mexico, Albuquerque, NM, United States

**Keywords:** tauopathy, degradation, transmission, tau, neuron, glial cells, autophagy, endo-lysosomal systems

## Abstract

Tauopathies are a class of neurodegenerative diseases, including Alzheimer’s disease (AD), Frontotemporal Dementia (FTD), Progressive Supranuclear Palsy (PSP), Corticobasal Degeneration (CBD), and many others where microtubule-associated protein tau (MAPT or tau) is hyperphosphorylated and aggregated to form insoluble paired helical filaments (PHFs) and ultimately neurofibrillary tangles (NFTs). Autophagic-endolysosomal networks (AELN) play important roles in tau clearance. Excessive soluble neurotoxic forms of tau and tau hyperphosphorylated at specific sites are cleared through the ubiquitin-proteasome system (UPS), Chaperon-mediated Autophagy (CMA), and endosomal microautophagy (e-MI). On the other hand, intra-neuronal insoluble tau aggregates are often degraded within lysosomes by macroautophagy. AELN defects have been observed in AD, FTD, CBD, and PSP, and lysosomal dysfunction was shown to promote the cleavage and neurotoxicity of tau. Moreover, several AD risk genes (e.g., *PICALM*, *GRN*, *and BIN1*) have been associated with dysregulation of AELN in the late-onset sporadic AD. Conversely, tau dissociation from microtubules interferes with retrograde transport of autophagosomes to lysosomes, and that tau fragments can also lead to lysosomal dysfunction. Recent studies suggest that tau is not merely an intra-neuronal protein, but it can be released to brain parenchyma via extracellular vesicles, like exosomes and ectosomes, and thus spread between neurons. Extracellular tau can also be taken up by microglial cells and astrocytes, either being degraded through AELN or propagated via exosomes. This article reviews the complex roles of AELN in the degradation and transmission of tau, potential diagnostic/therapeutic targets and strategies based on AELN-mediated tau clearance and propagation, and the current state of drug development targeting AELN and tau against tauopathies.

## Introduction

Microtubule-associated protein tau (MAPT or tau) is a neuronal protein, which binds to the microtubule and regulates its assembly and stabilization, thus mediating the axonal transport of cellular components along the microtubule. Tau is constantly phosphorylated and dephosphorylated under physiological conditions (Lee et al., 2001; Götz et al., 2019). However, under pathological conditions, tau undergoes various post-translational modifications and detaches from microtubule to form insoluble aggregates, named paired helical filaments (PHFs) that ultimately grow into neurofibrillary tangles (NFTs).

A variety of post-translational modifications and aggregation of tau and the presence of PHFs and NFTs inside neurons or glial cells (astrocytes and oligodendrocytes) is the pathological hallmark of AD and related tauopathies (Lee et al., 2001). Tau pathology positively correlates with the disease progression and aligns well with Braak staging (Schöll et al., 2016). Different tauopathies display different brain regional susceptibility and distinct clinical symptoms due to the underlying type and extent of tau pathologies (Götz et al., 2019).

The Ubiquitin-proteasome system (UPS) and autophagy are two major intracellular degradation process (Dikic, 2017). UPS mainly degrades short-lived proteins, and autophagy is responsible for the clearance of long-lived proteins and damaged organelles. Autophagy can be further categorized into chaperon-mediated autophagy (CMA), endosomal microautophagy (e-MI), and macroautophagy.

Soluble neurotoxic tau is cleared by UPS (Lee et al., 2013), CMA (Wang et al., 2009), and e-MI (Caballero et al., 2018; Uytterhoeven et al., 2018), while intracellular insoluble tau is degraded by macroautophagy (Krüger et al., 2012). In addition, tau phosphorylated at specific sites can also be removed by the autophagy-independent endolysosomal system (Vaz-Silva et al., 2018). Apart from AD/tauopathies, NFTs are also found in brains of subjects with Niemann-Pick type C (NPC), a lysosomal storage disease resulting from lysosomal function defects (Wang and Mandelkow, 2012). Thus, it suggests the Autophagy Endo-Lysosomal Network (AELN) plays an important role in preventing tau aggregation and NFT accumulation in different tauopathies and lysosomal storage diseases.

Indeed, accumulation of lysosomes, lysosomal hydrolases, autophagosome, autophagic vacuoles, multi-vesicular bodies (MVBs), autolysosomes, and defect in lysosomal membrane integrity in the brain has been reported in AD, CBD, and PSP patients (Nixon et al., 2005; Nixon, 2013; Piras et al., 2016; Menzies et al., 2017). Defects in the retrograde transportation of autophagosomes from axon to soma and antegrade transportation of degradative lysosomes to distal axons were also demonstrated (Farfel-Becker et al., 2019). On the other hand, fragmented tau can impair CMA and lysosomal dysfunction and lead to tau oligomerization and aggregation (Wang et al., 2009).

*BIN1*, *PICALM*, *CD2AP*, *RIN3*, *SORL1*, *GRN*, and *PLD3*, which affect AELN functions (Van Acker et al., 2019), were identified as AD susceptibility loci in genome-wide association studies (reviewed in Van Acker et al., 2019). Particularly, *BIN1*, *PICALM*, and *GRN* have been implicated in tauopathies. These linkage studies suggest the failure of AELN can contribute to tau pathology in tauopathies and related diseases.

Interestingly, AELN is also involved in the propagation of tau. Physiological and pathological tau can both be secreted from neurons through exosomes, the extracellular vesicles contained inside MVBs (an intermediate endocytic compartment) (Saman et al., 2011; Wang et al., 2017). MVB fusion with the plasma membrane releases exosomes to the extracellular space. Secretory autophagy-based unconventional secretion of pathological tau has also been reported in a recent study (Kang et al., 2019).

Uptake of different forms of tau by neurons utilizes AELN as well. Internalized tau then clogs up UPS and AELN, further inhibiting tau degradation and contributing to pathological tau accumulation (Wu et al., 2013). In addition, once internalized, tau aggregates can rupture endosomes to seed more tau aggregates and propagate in the recipient cells (Calafate et al., 2016).

Microglia can effectively phagocytose tau aggregates bound to anti-tau antibodies and degrade them through AELN (Luo et al., 2015). On the other hand, microglia can also be a foe in propagating tau pathology by packaging tau into exosome and release into extracellular space (Asai et al., 2015). Furthermore, astrocytes can take up tau fibrils through HSPG-mediated micropinocytosis (Martini-Stoica et al., 2018) and tau monomers through an HSPG-independent pathway (Perea et al., 2019). Upon internalization into astrocytes, astroglial transcription factor – EB (TFEB, a master regular of autophagy) promotes tau degradation and the inhibition of tau transmission in tauopathies (Martini-Stoica et al., 2018).

In this review, we will briefly discuss the pathways responsible for tau degradation, release, and uptake in neurons and glial cells. We will also summarize the potential therapeutic targets and those that are already in clinical trials for promoting tau degradation and preventing tau propagation.

## Structure and Function of Tau in Tauopathies

Tau is a microtubule-associated protein encoded by the *MAPT* gene. Alternative splicing of the single *MAPT* mRNA generates six different isoforms (Himmler et al., 1989). The longest tau isoform with two-amino terminal inserts and four microtubule-binding domains (2N4R-Tau) has more than 80 phosphorylation sites (Iqbal et al., 2016).

Under normal conditions, tau is constantly phosphorylated and dephosphorylated to regulate the microtubule assembly in axons. Tau is also involved in facilitating the axonal transport of cellular cargoes on microtubule tracks in neurons by differentially regulating two motor proteins, dynein and kinesin (Dixit et al., 2008). In healthy neurons, lower tau concentration in the soma enables kinesin’s binding to microtubules and anterograde transport of cargoes to the distal axon. Cargoes are then released due to the higher tau concentration at the distal axon. Whereas dynein-dependent retrograde transport from the axon to soma is not affected because of dynein’s lower sensitivity to tau. However, tau-dependent axonal transport is still controversial since it is not validated in *in vivo* studies. One study even shows tau overexpression or knockout in a mouse model does not show impairment in the axonal transport (Yuan et al., 2008).

Under pathological conditions, tau undergoes various types of post-translational modifications, including hyperphosphorylation and detaches from microtubules. Detached tau can either exist as soluble monomers or form neurotoxic oligomeric aggregates, paired helical filaments (PHFs), and ultimately neurofibrillary tangles (NFTs). Loss-of-function of tau, which causes disassembly of the microtubule, and gain-of-function of neurotoxic tau aggregates, have both been proposed during the disease progression of AD and related tauopathies (Rapoport et al., 2002; Trojanowski and Lee, 2005; Avila et al., 2010).

Tauopathies are a group of more than twenty diseases, where tau is hyperphosphorylated and aggregated to form intracellular inclusions NFTs inside neurons or glial cells (Götz et al., 2019). The most common forms of primary tauopathies are Pick’s disease (PiD), Progressive Supranuclear Palsy (PSP), Corticobasal Degeneration (CBD), and frontotemporal dementia with parkinsonism liked to chromosome 17 (FTDP-17). The secondary tauopathy, Alzheimer’s disease (AD), is the most prevalent form of tauopathy, affecting more than 50 million people worldwide and 50 million expected to increase to 152 million by 2050 (World Alzheimer Report, 2019). Familial tauopathies are caused by pointmutations in the *MAPT* gene or alternative splicing of *MAPT* mRNA – resulting in the imbalance in different tau isoforms, and/or numerous types of post-translational modifications. Accordingly, patients with tauopathies display different brain regional susceptibility and present with distinct clinical symptoms, which is driven by the underlying type and extent of tau pathologies. The molecular pathogenesis of tauopathies is extensively reviewed in another review (Götz et al., 2019).

## Clearance of Pathological Tau by Autophagy-Endolysosomal Network

**FIGURE 1:**
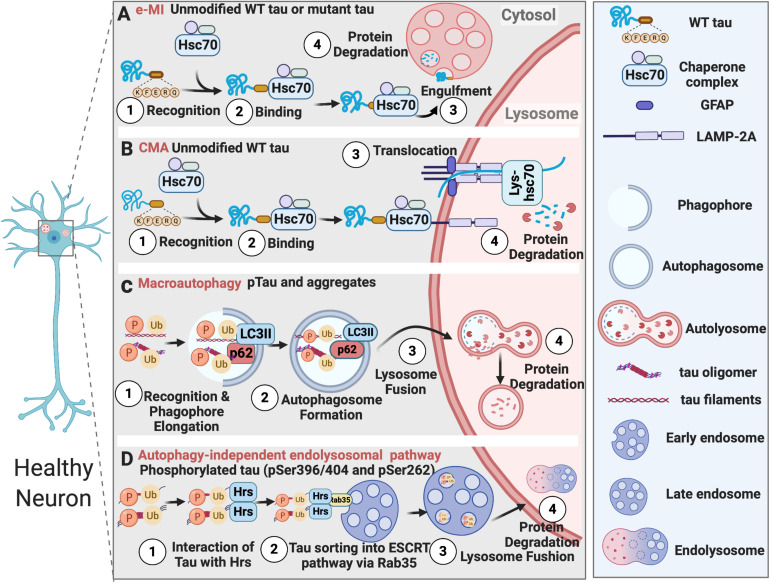
Tau degradation in healthy neurons. **(A)** Unmodified wildtype tau or mutant tau is degraded by e-MI; **(B)** Unmodified wildtype tau is degraded by CMA; **(C)** Phosphorylated tau and tau aggregates are degraded by macroautophagy; **(D)** Tau phosphorylated at specific sites is degraded by autophagy-independent endolysosomal pathway.

The ubiquitin-proteasome system (UPS), Chaperon-mediated Autophagy (CMA), and endosomal microautophagy (e-MI) clear soluble neurotoxic tau, while macroautophagy degrades intracellular insoluble tau inside neurons. Additionally, autophagy-independent endolysosomal degradation of ubiquitinated tau with certain phosphorylation sites has also been reported (Figure 1). Tau degradation by UPS is extensively reviewed elsewhere (Lee et al., 2013). Numerous studies have reported that all three different types of autophagy process – CMA (Wang et al., 2009), e-MI, and macroautophagy, participate in the endolysosomal degradation of pathological tau. In brains of AD subjects, Alz50-positive conformationally altered tau-containing intraneuronal granules exhibited lysosome-like structures in regions surrounding infarcted foci in the human cerebral cortex, suggesting a failure of lysosomal degradation in tau tangles (Ikeda et al., 1998, 2000). Aside from AD/tauopathies, the fact that NFTs are also found in brains of subjects with Niemann-Pick type C (NPC), a lysosomal storage disease resulting from lysosomal function defects, suggests endolysosomal failure might be inducing tau aggregation and tangle build-up in the brain (Wang and Mandelkow, 2012).

### CMA-Dependent Degradation of Pathological Tau

CMA is one of the first discovered processes, which can selectively degrade cytosolic components by lysosomes (Dice, 1982). It does not utilize any membrane structures to engulf cargo, but instead utilizes chaperone proteins, such as HSC70, HSP90, HSP40, to recognize cargo proteins that have a KFERQ motif. Then these cargoes are unfolded and translocated directly across the lysosomal membrane for degradation (Cuervo and Wong, 2014; Kaushik and Cuervo, 2018).

During physiological conditions (upon damage or partial unfolding), CMA regulates various biological activities, including DNA repair, glucose/lipid metabolism, cellular reprogramming, and response to stress, through mediating the turnover of cellular non-aggregated proteins that have KFERQ motifs. CMA failure can result in several neurodegenerative diseases, including Parkinson’s disease, AD, frontotemporal lobar degeneration (FTLD), amyotrophic lateral sclerosis, and Huntington’s disease. Neurodegeneration is often accompanied by the accumulation of CMA substrates like α-synuclein, carboxyl-terminal hydrolase isozyme L1 (UCHL1), Tau, and TAR DNA-binding protein 43 (TDP-43) (Cuervo and Wong, 2014; Kaushik and Cuervo, 2018).

CMA-dependent degradation of unmodified wild-type tau was first described by Wang et al. (2009). However, in an inducible mouse neuroblastoma N2a cell model of tauopathy expressing the repeat domain of Tau with an FTDP-17 mutation (Tau_RD_ΔK280), Tau_RD_ΔK280 can only be partially cleaved by a thrombin-like activity in the cytosol to generate the F1 fragment. F1 fragment has two CMA targeting motifs (^336^QVEVK^340^ and ^347^KDRVQ^351^) and can be targeted to the CMA receptor by the chaperone heat-shock cognate protein 70 (Hsc70) and bind to the lysosomal membrane via lysosomal associated membrane protein 2A (LAMP-2A). However, F1 fragment can only partially translocate into lysosomes. This promotes Cathepsin L-mediated cleavage of F1 fragment at the C terminus to generate F2 and F3 fragments and further induces Tau_RD_ΔK280 oligomerization and aggregation, which can be degraded through the autophagy pathway. The oligomers of Tau_RD_ΔK280 can further interfere with lysosomal function and lead to lysosome leakage (Wang et al., 2009).

### Macroautophagy-Dependent Degradation of Pathological Tau

Macroautophagy is a more complex and selective process than CMA. For the macroautophagy to occur, a double-membrane structure called autophagosome forms around protein aggregates, damaged organelles, and microbes prior to their engulfment. Macroautophagy substrates are recognized by selective autophagy receptors (SAR), such as p62/SQSTM1 (sequestosome-1), which binds to the mammalian ATG8 proteins anchored to the inner membrane of the autophagosome, through LC3-interacting region (LIR) motifs (Johansen and Lamark, 2020). Macroautophagy-dependent degradation of pathological tau and its failure in tauopathies have been reported in numerous studies (Wang and Mandelkow, 2012; Menzies et al., 2017; Lee et al., 2013).

It has been observed that hyperphosphorylated tau co-localizes with LC3-positive vesicles and p62 receptor protein in the post-mortem brains of familial AD, CBD, and PSP patients (Piras et al., 2016). Furthermore, in cultured primary neurons, macroautophagy, but not proteasome, was responsible for the degradation of endogenous tau (Krüger et al., 2012). Aligned with this observation, autophagy inhibition by chloroquine enhanced tau aggregation (Hamano et al., 2008), and autophagy induction with rapamycin reduced tau levels *in vitro* and *in vivo* in a Drosophila model expressing human tau. In Parkin-knockout and tau-overexpressing mice, trehalose, which stimulates autophagy, alleviated tau pathology (Rodríguez-Navarro et al., 2010). Pharmacological inhibition of phospholipase D1 (PLD1), which is associated with the endosomal system and located on the outer membrane of the autophagosome, by a small molecule 5-fluoro-2-indolyl des-chlorohalopemide (FIPI) in organotypic brain slices led to an accumulation of Tau and p62 aggregates (Dall’Armi et al., 2010). In summary, these studies suggest the involvement of macroautophagy in the degradation of tau aggregates.

### e-MI-Dependent Degradation of Pathological Tau

Tau is also degraded by another form of autophagy called microautophagy. Microautophagy was considered to be a non-selective autophagic process, which can internalize soluble cytosolic cargoes through invaginations of the lysosomal membrane. However, a selective form of microautophagy, which recognizes cargoes harboring KFERQ motifs with the help of Hsc70 and delivers them into late endosomes, was discovered and named endosomal microautophagy (e-MI) (Sahu et al., 2011). It has been shown that more than half of the synaptic proteins have KFERQ motifs and can be recognized by the chaperone protein Hsc70. They are delivered to late endosomes/multi-vesicular bodies (MVBs) and degraded in the *Drosophila* neurons. Therefore, e-MI regulates the turnover of old and damaged synaptic proteins and neurotransmitter release (Uytterhoeven et al., 2015). Interestingly, tau also has two KFERQ motifs, and inducing microautophagy reduced mutant tau levels and synaptic vesicle sequestration at the pre-synaptic terminal in the *Drosophila* neurons (Uytterhoeven et al., 2018).

As summarized above, all three forms of autophagy can mediate the degradation of tau. However, how these pathways differ or overlap is largely unknown. Caballero et al. (2018) studied two different tau mutations, P301L, which causes autosomal−dominant FTDP-17 (Hutton et al., 1998), and A152T that does not cause autosomal−dominant disease but associates with a higher risk of FTD and AD (Coppola et al., 2012). Interestingly, the degradation of these mutant forms of tau by three different forms of autophagy varied in N2a cells. A152T mutation disrupted tau degradation in late endosome by e-MI, while macroautophagy was able to effectively degrade A152T tau, and its degradation by CMA was only mildly affected. Whereas the upregulation of macroautophagy or CMA did not increase the degradation of P301L-tau or reduce its cytotoxicity, suggesting merely upregulating macroautophagy or CMA may not achieve the therapeutic goal in efficiently clearing autosomal-dominant forms of mutated tau. Nonetheless, CMA may be effective in clearing the non-autosomal-dominant type of tau relevant to FTD and AD. On the other hand, e-MI was only able to degrade wild-type tau, but not the mutant forms of tau, a concept needing further evaluation as a recent study (Uytterhoeven et al., 2018) contradicted this conclusion. These findings suggest different tau mutants may favor certain degradative pathways. The differential degradation of tau mutants should be taken into consideration when modulating autophagy for therapeutic intervention in diverse types of tauopathies.

### Autophagy-Independent Endolysosomal Degradation of Pathological Tau

Autophagy-independent endolysosomal degradation of tau was also reported recently in primary hippocampal neurons and N2a cells (Vaz-Silva et al., 2018). Intriguingly, tau was found in Hrs/EEA1/Rab5-positive early endosomes, MVB vesicles, LAMP1-positive vesicles, suggesting its trafficking through the entire endolysosomal pathway. Tau interacted with the initial endosomal sorting complex required for transport (ESCRT) protein Hrs, possibly through ubiquitination, since the interaction was increased by deubiquitylating enzyme inhibitors. Tau sorting into the ESCRT pathway was dependent on the small GTPase Rab35. Rab35-dependent degradation of tau was particularly specific to tau phosphorylated at specific sites. For example, pSer396/404 and pSer262, but not pSer202. This finding again suggested the preferential sorting of specifically phosphorylated tau by multiple AELN pathways.

## Autophagy-Endolysosomal Network Defects in Tauopathy

**FIGURE 2:**
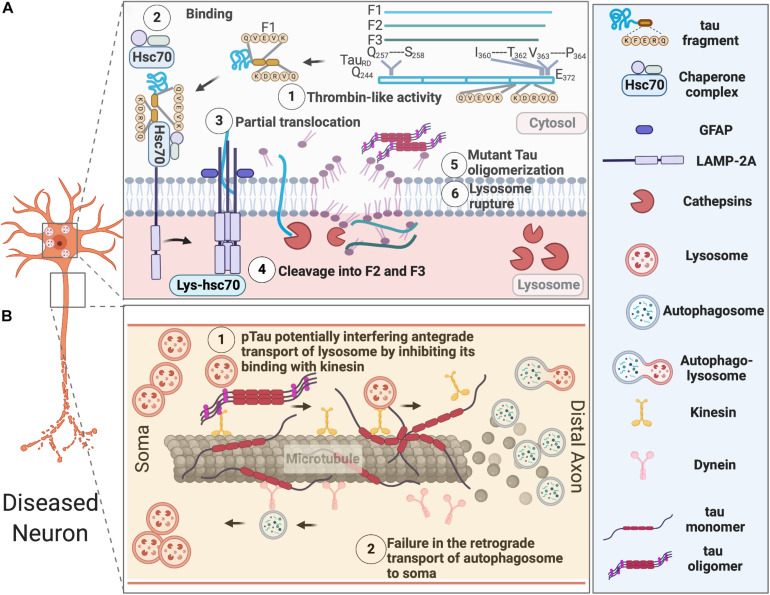
AELN defects in diseased neurons. **(A)** Exogenously expressed tau repeat domain can be cleaved by a Thrombin-like activity to generate an F1 fragment, which is then recognized by Hsc70 and co-chaperons. Hsc-70 bound F1 fragment can only partially translocate into lysosomes, which further induce its cleavage by Cathepsin L to generate F2 and F3 fragments. Tau fragments impair CMA and cause lysosomal rupture, which in turn induce mutant tau oligomerization and aggregation. **(B)** Pathological tau undergoes hyperphosphorylation and aggregates in the somatodendritic compartments, overwhelming AELN and also inhibits kinesin’s binding to the microtubules, which further interfere with the degradative lysosome transportation to axons. At the later stage, dissociation of hyperphosphorylated tau from microtubule and its destabilization/disassembly may interfere with the retrograde transport of autophagosomes to lysosomes and ultimately lead to accumulation of autophagosomes in axons.

Accumulation of lysosomes and lysosomal hydrolases and defects in lysosomal membrane integrity has been reported in AD patients (Perez et al., 2015). Increased levels of lysosomal components cathepsin D and LAMP1 was also found in the brains of CBD and PSP patients (Piras et al., 2016). The buildup of autophagosomes, autophagic vacuoles, MVBs, multilamellar bodies, and cathepsin-containing autolysosomes is also apparent in the dystrophic neurites in the brains of AD subjects (Nixon et al., 2005; Nixon, 2013; Menzies et al., 2017), which indicates defects in autophagosome maturation, transport, and fusion with lysosomes during the course of pathological progression (Figure 2).

In healthy neurons, autophagosomes are initially produced at the terminal of axons and transported retrogradely to the soma, where lysosomes are located. This retrograde transport of autophagosomes along the microtubule is dynein- and activity-dependent in granule cells (Katsumata et al., 2010). It was recently reported that soma-derived degradative lysosomes are anterogradely transported to the distal axon to fuse with autophagosomes to form autolysosomes, which are later retrogradely transported to the soma for degradation in mouse cortical neurons at DIV 14 (Farfel-Becker et al., 2019). In disease neurons, impaired axonal delivery of lysosome causes autophagic stress. Whether it contributes to tau pathology needs further investigation.

Additionally, lysosomal pH dysregulation and aberrant activity of a proton pump-the vacuolar ATPase (v-ATPase) have been implicated in various neurodegenerative diseases, including AD (Colacurcio and Nixon, 2016). Particularly, a brain proteomic analysis (Chang et al., 2013) revealed the expression of v-ATPase H+ Transporting V1 Subunit E1 (ATP6V1E1), which is responsible for the acidification of various intracellular organelles (endosomes, lysosomes, the trans-Golgi network, and synaptic vesicles), is altered throughout the course of tau tangle pathologies. This study utilized a transgenic mouse model (NSE-htau12) expressing human wild-type tau under the control of neuron-specific enolase (NSE). ATP6V1E1’s expression was increased in the early disease process, but decreased later, suggesting lysosomal function was augmented upon protein aggregate challenge, then overwhelmed and compromised later as the disease progresses.

## Genetic Risks Associated With the Autophagy-Endolysosomal System in Tauopathies

Genome-wide association studies have identified 27 AD susceptibility loci. Many of them (for example, *BIN1*, *PICALM*, *CD2AP*, *RIN3*, *SORL1*, *GRN*, and *PLD3*) affect AELN functions (Van Acker et al., 2019). Particularly, *BIN1*, *PICALM*, and *GRN* have been implicated in tauopathies.

### BIN1

Bridging Integrator 1 (BIN1) is a cytoplasmic protein involved in vesicle-mediated transport. It is localized on early and late endosomes, lysosomes, and recycling endosomes and can bind to tau, dynamin, and clathrin. In AD brains, neuronal isoform 1 of BIN1 protein level is decreased, and microglial isoform 9 is increased (Holler et al., 2014). Protein levels of BIN1 have been shown to negatively correlate with tau pathology in an *in vitro* model of BIN1 knockout rat hippocampal neurons, where accumulated tau disrupts endosomes and propagates to the cytosol to induce more aggregates. The underlying mechanism was BIN1 reducing the endocytic flux through Rab5 deactivation (Calafate et al., 2016).

### PICALM

Phosphatidylinositol Binding Clathrin Assembly Protein (PICALM) is a clathrin-assembly protein that can recruit the adaptor complex 2 (AP2) to clathrin-coated pits to regulate membrane cycling. PICALM is an AD risk gene, and single-nucleotide polymorphism (SNP) in PICALM leads to abnormal cleavage of PICALM by calpain and caspase-3 (Ando et al., 2013). It has been shown that PICALM modulates autophagy and its depletion leads to autophagic dysfunction, which contributed to the accumulation of phosphorylated tau in primary mouse neurons (Moreau et al., 2014). PICALM colocalizes with phosphorylated 3R and 4R tau in the brains of AD, FTLD, PiD, and PSP patients (Ando et al., 2016).

### GRN

The mutation of Granulin precursor (*GRN*), another risk gene for FTD besides *MAPT*, is also implicated in AD cohorts (Perry et al., 2013). Progranulin (PGRN), encoded by the human *GRN* gene, is a secreted growth factor that can localize to intraneuronal membrane compartments, including lysosomes. Homozygous GRN mutations can cause a rare lysosomal storage disease (Dickson et al., 2010; Paushter et al., 2018), suggesting a plausible interaction between PGRN and tau in contributing to FTDs.

In addition to the genes mentioned above, mutations in charged multivesicular body protein 2B (CHMP2B) (Skibinski et al., 2005), in the ESCRT-III complex, and C9ORF72, which colocalizes with RAB proteins, are linked to defects in endosomal trafficking in FTD (Farg et al., 2014).

## Tau Transmission

**FIGURE 3:**
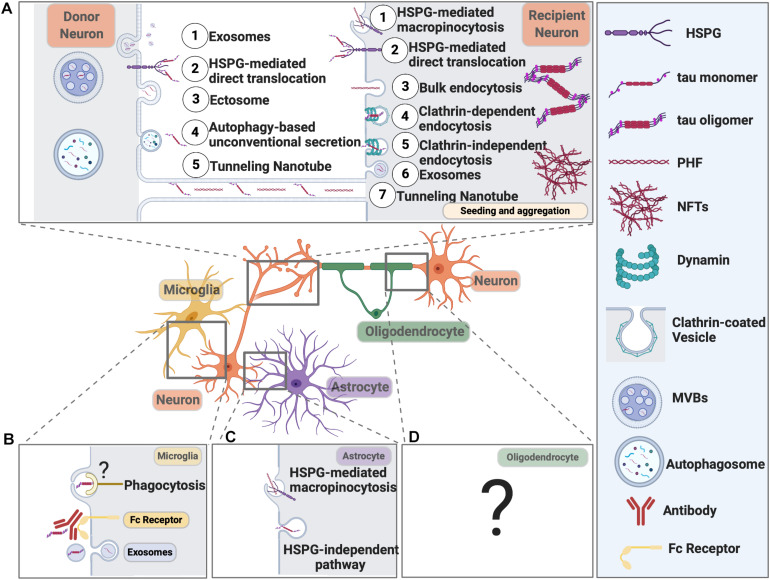
Pathways Mediating Pathological Tau Release and Uptake in Neurons and Glial Cells. **(A)** Neurons release pathological tau through the following pathways: (1) Exosomes; (2) HSPG-mediated direct translocation; (3) Ectosome; (4) Autophagy-based unconventional secretion; (5) Tunneling nanotube. Neurons can internalize pathological tau through the following pathways: (1) HSPG-mediated macropinocytosis; (2) HSPG-mediated direct translocation; (3) Bulk endocytosis; (4) Clathrin-dependent endocytosis; (5) Clathrin-independent endocytosis; (6) Exosomes; (7) Tunneling Nanotube. **(B)** Microglial cells internalize tau through exosome and phagocytosis. Microglia can also take up antibody-bound tau fibrils. Internalized tau can be degraded or packaged into exosomes and propagate. The receptor on the microglial cell surface that recognizes pathological tau is yet to be defined. **(C)** Astrocytes take up tau fibrils through HSPG-mediated macropinocytosis and tau monomers through an HSPG-independent pathway that needs further investigation. **(D)** The molecular pathways responsible for tau uptake in oligodendrocytes remain elusive.

Mounting evidence now suggests tau, together with many other proteins, involved in neurodegeneration, can propagate like prion from cell to cell either via direct physical synaptic connections or through extracellular vesicles, like exosomes and ectosomes, or by tunneling nanotubules, other endocytic pathways (extensively reviewed in Fuster-Matanzo et al., 2018; Gibbons et al., 2019; Uemura et al., 2020). Tau not only propagates between neurons, but it can also spread from neurons to microglia, astrocytes, and oligodendrocytes (Figure 3). It is speculated that tau might even spread from glial cells to glial cells in tauopathies like PSP and CBD (Narasimhan et al., 2017), eventually forming astrocytic plaques and/or tufted astrocytes.

Spreading of tau pathology to synaptically connected brain regions was initially suggested from the hierarchical pattern of tau pathology beginning in the trans-entorhinal cortex and eventually spreading to synaptically connected brain regions, such as the hippocampus and, later, the cortex (Braak and Braak, 1995). An *in vitro* study first showed that extracellular recombinant tau aggregates, but not monomers, were internalized by a mouse immortalized neural progenitor cell line C17.2 and induced fibrillization of intracellular full-length tau, which was able to further cross-seed fibrillization of recombinant tau monomer (Frost et al., 2009). Another *in vitro* study (Guo and Lee, 2011) reported the introduction of a small amount of misfolded preformed fibrils of tau (pffs) into QBI-293 immortalized human embryonic kidney cells transfected with human tau, turned a large amount of soluble Tau into NFT-like filamentous inclusions. Uptake of pffs into the cells was mediated by endocytosis, and the P301L mutation of tau enhanced pff-induced aggregation accompanied by reduced microtubule stability. Conclusions from these findings were also supported by multiple *in vivo* studies utilizing pathological tau from different resources in different mouse models.

Injection of brain extract from P301S human tau mice into the brains of ALZ17 transgenic mice expressing wild-type human tau induced seeding of wild-type human tau to become tau filaments and spreading of tau pathology from the injection site to the neighboring brain regions (Clavaguera et al., 2009). Mis-localization of tau from axons to somatodendritic compartments and spreading of tau pathology from the entorhinal cortex to the surrounding brain regions, including the subiculum, CA1, and dentate gyrus was also observed in an inducible mouse line Neuropsin-tTA-Tau (NT), which differentially express tau in the EC region (Liu et al., 2012) and mimics the Braak staging. Tau propagation is dependent on synaptic circuitry since in rTgTauEC mice, a transgenic mouse model exclusively expressing human P301L tau in the layer II of the entorhinal cortex (EC), tau pathology initiated from EC transgene-expressing neurons to non-transgene expressing neurons downstream in the synaptic circuit such as the dentate gyrus, hippocampus CA region, and cingulate cortex (De Calignon et al., 2012). Tau propagation theory was further supported by the evidence that synthetic tau pffs alone can seed endogenous tau to become NFT-like structures in a dose- and time-dependent manner in the interconnected brain regions of PS19 mice (Iba et al., 2013).

### Tau Release From Neurons

For tau to spread between cells, it first needs to be released from neurons. Studies have shown that in addition to being passively released from dead cells, tau can also be actively released from neurons in vesicle-associated form or non-vesicle-associated free form (Tables 1, 2).

**TABLE 1:** Pathways mediating physiological tau release from neurons.

**Pathways**	**Forms of Tau**	**Characteristics**	**References**
Non-vesicular secretion	Free form	Majority of Secreted tau is in free, non-vesicular form. Whether it is HSPG- dependent was not examined.	Dujardin et al., 2014
Non-exosome associated	Physiological tau, non-aggregating form	AMPA-mediated, calcium-dependent tau release enhanced with increased neuronal activity. Not clear if this pathway involves other vesicles.	Pooler et al., 2013
Ectosome	Dephosphorylated, full-length tau or C-terminus truncated	Found in mouse ISF samples, slightly switches to exosomal secretion when tau accumulates inside cells.	Dujardin et al., 2014; Cocucci and Meldolesi, 2015
Autophagy-based unconventional secretion	Total tau	Released into media without any stimulation in SHSY-5Y cells	Kang et al., 2019
			

**TABLE 2:** Pathways mediating pathological tau release from neurons.

**Pathways**	**Forms of tau**	**Characteristics**	**References**
Exosome	Total tau, pT181- tau, pS396-tau, fragmented tau lacking C-Terminus	Neuronal activity- and synaptic connectivity-dependent	Saman et al., 2011; Dujardin et al., 2014; Wang et al., 2017
Ectosome	Fragmented tau lacking C-Terminus	Slightly switches to exosomal secretion when tau accumulates inside cells.	Dujardin et al., 2014
Tunneling Nanotube	Exogenous tau monomers and fibrils	Filamentous-actin-containing membranous structures	Tardivel et al., 2016
HSPG- mediated direct translocation	Soluble full-length tau, phosphorylated, oligomeric	Secreted soluble tau utilized the same HSPG-mediated direct translocation mechanism to transcellularly enter the recipient cells and induced aggregation	Katsinelos et al., 2018; Merezhko et al., 2018
Autophagy-based unconventional secretion	Various forms of pTau	Nigericin induced	Kang et al., 2019

Exosomes are extracellular vesicles, which are formed inside an MVB. Exosome release into the extracellular space is dependent on the fusion of the MVB with the plasma membrane. Secreted tau in the supernatant of M1C neuroblastoma cells was exosome-associated and mostly enriched for pT181 (AT270 antibody site), an established early AD biomarker used in CSF-based AD diagnosis. The authors also found that CSF samples from mild AD patients had an increased level of exosome-associated pT181 tau (Saman et al., 2011). The exosome-mediated release and trans-synaptic transmission of Tau between neurons are dependent on neuronal activity and synaptic connectivity, as shown by an *in vitro* microfluidic study (Wang et al., 2017). Interestingly, exosomal tau was taken up by neurons and microglia, but not astrocytes, and microglia was more efficient in internalizing exosomal tau. Exosomal tau released from N2a cells or monomeric or oligomeric tau from exosomes derived from AD patients CSF samples can both induce tau aggregation in cultured cells.

Besides exosomes, another type of vesicles, ectosomes (Cocucci and Meldolesi, 2015), is also shown to be involved in tau secretion. Ectosomes vesicles (0.1–1 μm in diameter) that are budded and released directly from the plasma membrane upon elevated levels of intracellular calcium, inflammatory molecules, or oxidative stress. One study showed that in addition to the major non-vesicular free form of tau is predominantly secreted in ectosomes *in vitro* and *in vivo* under physiological conditions (Dujardin et al., 2014). Ectosomal tau was also found in the interstitial fluid (ISF) of mice in the same study. This physiologically secreted tau is mainly not phosphorylated. However, intracellular pathological accumulation of tau leads to a slight shift toward tau secretion by exosomes (Dujardin et al., 2014). Interestingly, exo- and ecto-somal tau appears to be lacking the C-terminus, indicating tau truncation (Guillozet-Bongaarts et al., 2007), which is another sign of tau pathology. Some important questions remain to be elucidated: (1) whether pathological tau secreted via ectosomes can be internalized and induce tau seeding in neurons; (2) whether ectosomal tau can be detectable in biological fluids (ISF, CSF, or blood) of tauopathy patients; (3) what are the molecular pathway(s) responsible for ectosomal tau release.

The third mechanism, Heparan sulfate proteoglycan (HSPG)-mediated direct translocation across the plasma membrane, was responsible for the secretion of soluble full-length tau *in vitro* in CHO cells with monomeric tau binding to PI(4,5)P2-containing liposomes, which resemble the inner leaflet of the plasma membrane (Katsinelos et al., 2018). The secreted soluble tau utilized the same HSPG-mediated direct translocation mechanism to transcellularly enter the recipient cells and induced aggregation of the GFP-tagged tau repeat domain (RD-GFP). Another study reported when overexpressing 0N4R isoform of tau in N2a cells led to the clustering of phosphorylated, oligomeric tau at the plasma membrane and then penetrate the plasma membrane to be secreted to the extracellular space, partially mediated by HSPG (Merezhko et al., 2018).

Exogenous tau monomers and fibrils are also reported to increase the number of tunneling nanotubes (TNTs), filamentous-actin-containing membranous structures that bridge and connect cells, which can facilitate the intercellular transfer of tau between neurons (Tardivel et al., 2016). However, the exact molecular mechanism of the TNT formation remains elusive, and the relevance of this occurring *in vivo* needs further investigation.

Interestingly, stimulating neuronal activity or inducing the α-amino-3-hydroxy-5-methyl-4-isoxazolepropionic acid receptor (AMPA receptor) with its agonist (*S*)−AMPA prompted tau release from healthy, mature primary cortical neurons (Pooler et al., 2013). In contrast, suppressing presynaptic vesicle release by tetanus toxin or inhibiting neuronal activity with tetrodotoxin both significantly blocked AMPA-mediated, calcium-dependent tau release. However, this mode of tau secretion was not associated with exosome, suggesting physiological tau release might differ from the unconventional secretion of pathological extracellular tau. This observation was confirmed by a subsequent *in vivo* study where the authors used *in vivo* micro-dialysis and found neuronal activity rapidly increases the preexisting tau in brain interstitial fluid (ISF) within hours in a transgenic mouse expressing a non-aggregating form of human tau (Yamada et al., 2014). However, the clearance of ISF tau was not rapid and suggested to take a longer time, maybe several days. Notably, this study did not examine other pathways involved in tau secretion, for example, HSPG-mediated direct translocation, ectosomes, and TNTs. Therefore, the involvement of other tau secretory pathways for clearing ISF-tau cannot be completely ruled out.

Similar to physiological tau release, pathological tau release was also enhanced with increased neuronal activity both *in vitro* and *in vivo*. Endogenous full-length tau derived from donor neurons from rTg4510 mice – expressing human P301L tau, was released into the extracellular media (although vesicle-associated or not is unclear) and taken up by recipient neurons to form tau aggregates. This pathological tau further induced neurofibrillary tangle formation in distant cells (Wu et al., 2016). Neuronal activity induction by an optogenetic approach increased tau release and cell-to-cell propagation *in vitro*. Optogenetically inducing neuronal activity also enhanced tau pathology and hippocampal cell layer atrophy in the stimulated hemisphere compared to the unstimulated hemisphere of rTg4510 mice. This finding was confirmed by using a chemogenetic approach to induce neuronal activity in the EC of a separate mouse line called “EC-tau.” Besides, tau released from human iPSC-derived neurons was also able to transfer to recipient donor neurons through the extracellular space. Although non-mutant tau, either from wildtype mice or from non-mutant human iPSCs, can also be released and internalized. Yet, this tau did not form neurofibrillary tangles even with prolonged time of culturing, suggesting the phosphorylated, aggregated tau or mutant tau can more easily accumulate and seed in the recipients rather than the soluble, non-mutant form of tau.

Apart from the degradation of intracellular components, autophagy has also been shown to regulate the ER- and Golgi-independent unconventional secretion of several leaderless proteins, like cystic fibrosis transmembrane conductance regulator (CFTR) (Gee et al., 2011), IL-1β (Dupont et al., 2011), and High Mobility Group Box 1 (HMGB1) (Thorburn et al., 2009). A recent study (Kang et al., 2019) suggested autophagy-based unconventional secretion of tau. Authors treated SHSY-5Y human neuroblastoma cells with bacterial toxin nigericin, which is also a potassium ionophore that can accumulate autophagic vesicles by raising the pH of acidic cellular compartments. They found both total tau and various types of phosphorylated tau are secreted into the supernatant, which can be inhibited through autophagic protein beclin1 knockdown. This tau secretion process took place without cell death. However, important questions still exist. For example, (1) whether Golgi reassembly-stacking protein of 55 kDa (GRASP55), an important protein involved in autophagy-based unconventional secretion of mammalian leaderless proteins, is involved in the autophagy-based tau secretion; (2) whether autophagy proteins are present in the same supernatant; and (3) whether tau is secreted in the same way in human primary microglia and *in vivo*.

## Tau Uptake by Neurons, Microglia, and Astrocytes

Secreted Tau can be internalized by recipient neurons, microglia, astrocytes, and oligodendrocytes via various mechanisms, including HSPG-mediated direct translocation, HSPG-mediated endocytosis, exosome-based uptake, tunneling nanotube, clathrin-independent endocytosis, clathrin-dependent endocytosis, and HSPG-dependent macropinocytosis.

### Uptake Into Neurons

Most studies have been focused on the uptake of tau into neurons. Neurons can internalize tau through HSPG-mediated direct translocation, exosomes, bulk endocytosis, tunneling nanotube, as mentioned above. Additionally, Clathrin-independent endocytosis and HSPG-dependent macropinocytosis are also shown to be involved in the internalization of Tau by neurons (Table 3).

**TABLE 3:** Pathways mediating pathological tau uptake into neurons.

**Pathways**	**Forms of tau**	**Recipient cells**	**Subsequent activities inside the recipient cell**	**References**
Exosome	Monomeric or oligomeric tau from exosomes derived from AD patient CSF, pT181 tau	Neuron	Seeding and Propagation	Saman et al., 2011; Wang et al., 2017
Clathrin-independent endocytosis	Monomeric tau	Neuron	Seeding and inducing aggregates, rupture endosome to activate Galectin8-NDP52 mediated autophagic degradation of monomeric tau	Falcon et al., 2019
HSPG-mediated direct translocation	GFP-tagged tau repeat domain	Neuron	Seeding and Propagating	Katsinelos et al., 2018; Merezhko et al., 2018
Bulk endocytosis	LMW tau aggregates and short fibrils	Neuron	Clogging up UPS and AELN	Wu et al., 2013
Tunneling nanotube	Exogenous Tau monomers and fibrils	Neuron	Unknown	Tardivel et al., 2016
HSPG-dependent macropinocytosis	Full-length tau fibrils	Neuron	Seeding and Propagating	Holmes et al., 2013; Puangmalai et al., 2020
Clathrin-dependent endocytosis	Exogenous TauP301L-GFP aggregates	Neuron	Rupture endosome to propagate, which can be inhibited by neuronal BIN1 isoform	Calafate et al., 2016

Full-length tau fibril uptake into neurons was macropinocytosis-dependent as HSPG inhibition blocked tau uptake and aggregation both in primary hippocampal neurons and in WT mice (Holmes et al., 2013). Another study has shown that exogenously added recombinant tau can misfold to form low molecular weight (LMW) aggregate, which can further assemble into short fibrils. Both LMW tau aggregates and short fibrils can be taken up by neurons through bulk endocytosis, but not monomers, long fibrils, nor long filaments (Wu et al., 2013). The internalization takes place either at the axon terminals and then tau being retrogradely transported to the cell body or happens at the somatodendritic compartment and then tau being delivered to the distal terminal axons. Internalized tau aggregates co-localize with late endosomes and lysosomes and potentially overload the proteasome and AELN system. It has been shown that exogenous tau can also be internalized via clathrin-dependent endocytosis and rupture endosome to propagate, which is negatively regulated by neuronal isoform 1 of BIN1 by inhibiting Rab5 and endosome formation *in vitro* (Calafate et al., 2016).

Autophagy is also linked to the degradation of monomeric tau internalized via clathrin-independent endocytosis within neurons, which further prevent tau seeding (Falcon et al., 2018a). To be specific, fluorescently labeled monomeric and assembled recombinant P301S tau was taken up by HEK293T or SH-SY5Y cells expressing P301S 1N4R tau. This uptake was mediated through clathrin-independent endocytosis. Internalized tau induced seeded aggregation of tau and further provoked the formation of tau-containing vesicles positive for Rab5 (endosomal marker) and Galectin-8, suggesting tau aggregates ruptured endosome, which can be detected by Galectin-8. Monomeric tau seeds were positive for nuclear dot 52 KDa protein (NDP52), and seeded tau aggregates were positive for p62, suggesting autophagy is activated to degrade the monomeric tau seeds and seeded tau aggregates through different autophagic receptors. siRNA knockdown of autophagosome components LC3C (also the specific binding partner of NDP52) and FAK family kinase-interacting protein of 200 kDa (FIP200) further increased seeded tau aggregation. On the other hand, expressing NDP52 lacking galectin-8–binding (NDP52L374A), LC3C-binding (NDP52V136S), or the SKICH domain (NDP52Δ1–126) was not able to reduce seeded tau aggregation (Falcon et al., 2018a). This finding indicates that NDP52-dependent autophagic degradation of monomeric tau seeds through galectin-8 is important in limiting the seeding and propagation of pathological tau.

A previous study showed that aberrant activation of p300/CBP, which acetylates tau, promotes its aggregation and inhibits its degradation by the proteasome, was observed in tauopathies (Min et al., 2010). The following study reported an additional role of p300/CBP in impairing autophagy-lysosomal pathways and increasing tau secretion in neurons. Overexpression of p300/CBP significantly increased the secretion of total tau and AT8-positive phosphorylated tau by inhibiting the autophagy flux *in vitro*, while inhibition of p300/CBP reduced synthetic tau fibril-induced seeding activity *in vitro* and *in vivo*. A new small inhibitor of p300/CBP, which can reduce tau secretion, was also identified through a high throughput screening (Chen et al., 2020). However, this study did not specify how p300/CBP is increasing tau secretion and which secretory pathway it utilizes. These studies emphasize the important roles of macroautophagy in degrading intracellular total tau, phosphorylated tau, internalized monomeric tau, and synthetic tau fibrils and further preventing the secretion and seeding of pathological tau.

In addition to tau uptake into neurons, glial tau pathology is common in various tauopathies. However, how glial cells internalize tau and what happens subsequently during healthy and disease conditions are still unclear. Table 4 summarizes the current state of knowledge about tau uptake by glial cells.

**TABLE 4:** Pathways mediating pathological tau uptake into glial cells.

**Pathways**	**Forms of tau**	**Recipient cells**	**Subsequent activities inside the recipient cell**	**References**
Exosome	Pre-aggregated human tau oligomers, hyperphosphorylated, oligomeric pT181 tau	Microglia	Release through exosomes and seeding, microglial BIN1 isoform 9-mediated	Asai et al., 2015; Wang et al., 2017; Crotti et al., 2019
Phagocytosis	Anti-tau antibody bound aggregated tau	Microglia	Degradation	Luo et al., 2015
HSPG-dependent Macropinocytosis	Pre-formed tau fibrils	Astrocytes	Degradation by macroautophagy	Martini-Stoica et al., 2018
HSPG-independent pathway	Exogenous tau monomers	Astrocytes	Unknown	Perea et al., 2019

### Uptake Into Microglia

As the major immune cells in the brain, microglial cells are important for the maintenance of homeostasis of the brain by clearing toxic substances by phagocytosis, immune surveillance, engulfing synapses, and mediating neurotransmitter turnover. Regarding tau, microglia can engulf tau aggregates bound to the anti-tau antibody, which is an important underlying mechanism of action for the tau passive immunization studies.

However, microglia can be a foe in spreading tau pathology and exacerbating the disease. Activating microglia in the hTau mouse model by knocking out *Cx3cr1*, the microglial fractalkine receptor (CX3CR1) that inhibits microglial activation, significantly increased AT180 tau hyperphosphorylation signal in the neurons from subiculum at 24 months of age, which correlated with CD45-positive microglia. Whereas at 2 months of age, only the CA1 region had AT180-positive neurons and CD45-positive neurons. These data suggest microglia activation might drive the spatiotemporal, anterograde-based spread of tau pathology in the anatomically connected regions of the hippocampus, from CA1 to subiculum (Maphis et al., 2015).

It has been shown that microglia can propagate pathological tau in the exosome-associated form (Asai et al., 2015). Depleting microglia in an AAV-based rapid human tau P301L propagation mouse model drastically suppressed tau propagation. Cultured primary mouse microglia were able to efficiently phagocytose exogenously added pre-aggregated human tau oligomers and also secrete exosomes containing ubiquitinated tau. This purified exosomal tau secreted from microglia was fluorescently labeled and stereotactically injected to the brains of wild-type mice. They were found to propagate inside the brain from the injection site to other brain regions, while exosomes with no tau or equal amount of soluble tau did not transmit inside the brain (Asai et al., 2015).

Microglial BIN1 has also been implicated in tau spreading. Unlike neuronal BIN1 isoform 1, which inhibits RAB5 activation and endosome formation to reduce tau propagation inside neurons, microglial BIN1 isoform 9 is shown to promote tau propagation by incorporating tau into exosomes (Crotti et al., 2019). BIN1 and hyperphosphorylated, oligomeric pT181 tau co-existed from the extracellular vesicles (EVs) containing exosomes purified from CSF samples of AD patients. BIN1-associated, tau-containing EVs induced seeding in an *in vitro* Tau-FRET assay. Overexpression of the ubiquitous BIN1 isoform 9 induced EV-associated tau release *in vitro* while the exclusively neuronal BIN1 isoform 1 did not. AAV-mediated overexpression of BIN1 isoform 9 in the hippocampus of PS19 mice worsened tau pathology, whereas BIN1 isoform 1 overexpression did not change pTau immunoreactivity. Knocking out microglial Bin1 from mice resulted in a significant reduction of tau in the EVs, suggesting microglial BIN1 is involved in the “packaging” of tau into the EVs. Conditional knockout of microglial *Bin1* in PS19 mice significantly reduced tau spreading compared to its *Bin1*-expressing littermates. These observations suggest that microglial BIN1 isoforms can contribute to the release of EV-associated tau during tau pathology (Crotti et al., 2019). This study provides another evidence that microglial cells can internalize and propagate pathological tau potentially through the exosomal/endosomal system.

### Uptake Into Astrocytes

Even though astroglial tau pathology is commonly seen in various tauopathies, the exact mechanisms of how astrocytes internalize pathological tau and the subsequent cellular processes after internalization are still elusive. Very few studies have investigated astroglial uptake of tau. It has been shown that pffs of tau can be internalized by astroglial cells through HSPG-mediated micropinocytosis (Martini-Stoica et al., 2018) (discussed in detail in the TFEB section below). Exogenously added tau monomers are reported to be taken up by astrocytes through an HSPG-independent pathway yet needs to be defined (Perea et al., 2019).

### Uptake Into Oligodendrocytes

A recent study injected brain homogenates from subjects with sporadic AD, primary age-related tauopathy (PART), pure aging-related tau astrogliopathy (ARTAG), globular glial tauopathy (GGT), PSP, PiD, and frontotemporal lobar degeneration linked to P301L mutation (FTLD-P301L) into the ipsilateral corpus callosum of adult wildtype mice induced tau seeding of murine tau in the contralateral corpus callosum (Ferrer et al., 2019). Phospho-tau deposits were found mainly in oligodendrocytes. However, the exact molecular mechanism mediating tau uptake into oligodendrocytes was not studied.

### Propagation and Seeding Capacity of Different Tau Strains

Still, the jury is out on whether monomeric, oligomeric, or fibrillar tau is the most toxic form, a lot of research focus has been on studying the propagation and seeding capacity of tau with different forms and different strain composition. Several studies now have shown that the conformation and size of tau aggregates determine its uptake. Short tau filaments seem to have the greatest seeding capacity.

Pathological tau indeed acts like prion that stably expressed tau repeat domain (RD) can propagate in a clonal fashion in cell cultures. The introduction of this tau RD into naïve cells can produce identical clones. Different tau strains can induce distinctive tau pathologies in naïve P301S mice, which can be steadily detected in three generations of tau inoculated P301S mice (Sanders et al., 2014). Of notice, by using the monoclonal Tet Off-tau RD-YFP cell line system, the authors found that tau RD strains derived from the brain homogenates from 29 patients with AD, Argyrophilic grain disease (AGD), PiD, and PSP displayed distinctive strain compositions across the diseases (Sanders et al., 2014). AD strains showed notable homogeneity, while most non-AD patient samples produced two or more strains with varying ability to cross-seed, which suggests the phenotypic diversity in the tauopathies (Sanders et al., 2014).

AD-patient derived tau fibrils also exhibited differential seeding potency and distinct conformational features from the synthetic, overexpressed tau fibrils, as shown in an *in vivo* seeding and propagation study in non-transgenic mice (Guo et al., 2016). Indeed, cryo-electron microscopy (cryo-EM) studies revealed that the heparin-induced tau polymers are different from those from AD or PiD, which have larger cores with different repeat compositions (Zhang et al., 2019), suggesting the complexities of tau fibrilization and seeding abilities in different tauopathies.

The differential seeding capacity of different tau strains or synthetic versus human brain-derived tau fibrils to form distinct aggregates and propagate may be related to the ultrastructural polymorphs of tau aggregates in different diseases. Recent cryo-EM studies have found that tau fibrils from different tauopathies present unique conformers. While AD and chronic traumatic encephalopathy (CTE) have distinct tau filaments contain both 3R and 4R tau (Fitzpatrick et al., 2017), tau filaments from PiD consist of residues Lys254–Phe378 of 3R tau (Falcon et al., 2018b) and those from CBD have four-layered fold with a large non-proteinaceous density (Zhang et al., 2020). *In vitro* ultrasensitive cell-free tau seed amplification assay also showed AD brain and CTE brain had seeding capacities that were orders of magnitude higher than those from PiD brains (Kraus et al., 2019). A very recent study examined neuronal internalization of soluble tau aggregates (oligomers) derived from brains of patients with AD, PSP, and Dementia with Lewy bodies (DLB). They found HSPG-mediated micropinocytosis is responsible for tau uptake in AD and DLB conditions, while the HSPG-mediated micropinocytosis and other pathways in other tauopathies are yet to be defined (Puangmalai et al., 2020). This study further suggested preferential uptake of different tau strains by different pathways, which further affect seeding and propagation of tau.

## Ameliorating Tau Pathology Through AELN Modulation by Targeting TFEB, the Master Regulator of Autophagy and Lysosome Biogenesis

Transcription factor EB (TFEB) is a master activator for the expression of many lysosomal genes (*ATP6V1H*, *ATP6V0E1*, *ATP6V1E1*, and *LAMP1*) and genes related to lysosome biogenesis and function (*CSTB*, *M6PR*, *IGF2R2009*, and *CTSD*), and autophagy genes (*SQSTM1*, *MAP1LC3B*, *UVRAG*, and *ATG9B*) (Sardiello et al., 2009; Settembre et al., 2011; Chauhan et al., 2013). TFEB is expressed in neurons, microglial, and astroglial cells. In human FTD and CBD brains, transcriptional levels of TFEB and LAMP1, protein levels of LAMP1, and cathepsin D (CTSD) increased compared to normal controls (Martini-Stoica et al., 2018). Transcriptional levels of human brain TFEB also positively correlated with cognitive decline in non-demented, mild cognitive impairment, and demented individuals (Martini-Stoica et al., 2018).

Various studies have shown that TFEB induction can reduce tau pathology and improve behavioral and synaptic functions both *in vitro* and *in vivo*. Adeno−associated virus (AAV)-TFEB injection into the lateral ventricles of both cerebral hemispheres on postnatal day 0 (P0) of rTg4510 mouse brains reduced neurofibrillary tangle pathology and reversed behavioral and synaptic deficits compared to their wildtype littermate controls when examined at 4-month post-injection time point (Polito et al., 2014). TFEB particularly degraded soluble and aggregated tau through autophagy and lysosomal activity by upregulating autophagy proteins (LC3B and p62) and lysosomal proteins (LAMP1 and CTSD), more specifically targeting phosphatase and tensin homolog (PTEN) *in vitro* (Polito et al., 2014). Overexpressing TFEB in another mouse model – P301S/flag-TFEB double-transgenic mice enhanced autophagy, restored neuronal loss, reduced PHF tau, reversed age-related deposition of lipofuscin granules, and rescued spatial, working, and reference memories (Wang et al., 2016).

High-content screening of FDA-approved drugs library identified two drugs, Flubendazole and Bromhexine. Bromhexine’s effect might be attributed to its metabolite ambroxol, which is linked to TFEB activation. Both drugs act as autophagy inducers through activating TFEB’s nuclear translocation by disrupting dynamic microtubule that leads to mTOR deactivation and dissociation from lysosomes (Chauhan et al., 2015). Interestingly, both drugs cleared S202 and S396/S404 hyperphosphorylated tau *in vitro* in N2a cells transfected with 0N3R-T231D/S235D phospho-mimicking human tau followed by treatment with conditioned media from RAW macrophages primed with LPS to induce hyperphosphorylation of tau. The clearance of pathological tau by these two drugs was autophagy-dependent, since knocking down Beclin1, a key autophagy protein, resulted in hyperphosphorylated tau accumulation. Considering the low toxicity of Flubendazole, further investigation should be completed in *in vivo* tauopathy models. Another study (Currais et al., 2014) discovered the pathological tau reducing effect of Fisetin, an organic flavonoid contained in many types of fruits and vegetables, can mitigate learning and memory deficits in APPswe/PS1dE9 double transgenic AD mice. Fisetin treatment in mouse primary cortical neurons and rat primary cortical neurons reduced S262 and S396/S404 hyperphosphorylated tau, decreased sarkosyl-insoluble tau in 293T cells co-transfected with plasmids expressing tau and constitutively active HA-GSK-3β-S9A. Fisetin-mediated pathological tau degradation was autophagy-dependent since chemical inhibitors of the autophagy-lysosome pathway decreased tau degradation. Its molecular mechanism of action was also identified as fisetin-mediated mammalian target of rapamycin complex 1 (mTORC1) inhibition, which leads to activation of TFEB and nuclear factor erythroid 2–related factor 2 (Nrf2), two transcription factors important for autophagy induction (Currais et al., 2014). A novel small molecule, which can activate TFEB, was also identified as Curcumin Analog C1 (C1) (Song et al., 2020). C1 effectively activated TFEB through direct binding, increased autophagy-lysosome activity, decreased amyloid pre-cursor protein (APP), APP C−terminal fragments (CTF−β/α), β−amyloid peptides, and tau aggregates respectively in 5xFAD, P301S, and 3xTg-AD mice, and also improved synaptic and cognitive function (Song et al., 2020).

The possibility of Opto-therapeutics to treat AD and other related tauopathies was demonstrated in a new study (Binder et al., 2020) that showed optical stimulation upon blue-light (465 nm) illumination can lead to TFEB expression, translocation into the nucleus, and activation, which can further induce autophagy and lysosomal gene expression and ultimately clear pathological Tau from neuronal cells and iPSC-differentiated neurons from an AD patient.

Intriguingly, a cell-specific contribution of TFEB in attenuating extracellular tau propagation and upregulating extracellular tau degradation was attributed to astrocytes both *in vivo* and *in vitro* (Martini-Stoica et al., 2018). AAV-TFEB overexpression in primary mouse astrocytes not only boosted the expression levels of lysosomal proteins, LAMP1, cathepsin B (CTSB), and CTSD but also increased the uptake of synthetic tau fibrils of the truncated form of human tau with the P301L mutation (K18 P301L). TFEB-enhanced uptake of preformed fibrils of tau (pffs) by astroglial cells was through HSPG-mediated macropinocytosis, which was reduced by heparin treatment. pffs internalized by astroglial cells were delivered into lysosomes and degraded. *In vivo* studies showed that AAV-TFEB induction in astrocytes in a slowly progressing PS19 tauopathy mouse model reduced tau pathology and activation of glial cells, while this was not seen in the more aggressive rTg4510 model. In a pff-induced NFT transmission mouse model, pffs were injected into the ipsilateral side of the brain. Astroglial TFEB expression reduced tau pathology in the contralateral hippocampus at the early time point and in both ipsilateral and contralateral hippocampi at the later time point. These findings suggest astroglial TFEB plays an important role in the upregulation of extracellular tau degradation and the inhibition of tau transmission in tauopathies (Martini-Stoica et al., 2018).

## Therapeutic Perspectives of Tau Degradation and Propagation by AELN

Understanding the molecular mechanisms that mediate the degradation and transmission of tau by AELN has a significant impact on the therapeutic interventions for different Tauopathies (Nixon, 2013; Li and Götz, 2017; Boland et al., 2018). The therapeutic strategy can be generally divided into two large categories: (1) Enhancing Tau degradation by AELN. (2) Preventing Tau Transmission through AELN.

### Enhancing Tau Degradation by AELN

Dozens of drugs that can enhance tau degradation through AELN have been identified and tested *in vitro* and/or *in vivo*, as summarized in Table 5. Of notice, cilostazol, nilotinib, and curcumin are already in Phase II Clinical Trials.

**TABLE 5:** Approaches to enhance tau degradation by AELN.

**Category**	**Name of interventions**	**Affected pathways**	**Mechanism of action**	**Models tested**	**Clinical trials**	**References**
Cathepsin inhibitors	EA-1	Lysosomes, tau cleavage, potentially CMA	Inhibits cathepsin D, which is involved in the lysosomal dysfunction and notably in the cleavage of the tau protein into tangle-like fragments	Organotypic hippocampal slice cultures	N/A	Bi et al., 2002
Chaperone modulators	VER-155008	CMA	Hsc70 inhibitor, reduces PHF-tau, reverses axonal degeneration, recovers memory function. Possibly inhibiting CMA and activating other tau degrading pathways	5XFAD mice	N/A	Yang and Tohda, 2018
Autophagy inducers	Lithium	Autophagy	Induces autophagy and inhibits GSK-3β-mediated tau phosphorylation	P301L Tau mice	Currently in AD Phase II clinical trial, NCT02129348	Shimada et al., 2012
Autophagy inducers	Trehalose	Autophagy	mTOR-independent autophagy inducer, activates AMPK and clears mutant tau	Primary neurons, P301S MAPT transgenic mouse	N/A	Schaeffer and Goedert, 2012
Autophagy inducers	Cilostazol	Autophagy	Upregulates Beclin 1, ATG5, LC3, and lysosomal Cathepsin B and downregulates mTORC1 and p300, and reduced tau acetylation and phosphorylation	N2a cells	Clinically approved for vascular disease, currently in AD Phase II clinical trial, NCT02491268	Lee et al., 2014
Autophagy inducers	Methylene blue	Autophagy	Inhibits tau oligomerization and promotes autophagy	Organotypic hippocampal slice/neurons and JNPL3 transgenic mouse with human P301L tau	Finished four Phase III clinical trials: NCT01689246 NCT01689233 NCT01626378 NCT02245568 Ongoing: NCT02380573	Congdon et al., 2012; Gauthier et al., 2016; Wilcock et al., 2018; Schelter et al., 2019; Shiells et al., 2020
Autophagy inducers	Temsirolimus	Autophagy	Reduced the accumulation of phosphorylated tau	SH-SY5Y cells and P301S tauopathy mice	N/A	Jiang et al., 2014
Tyrosine-protein kinase ABL1 inhibitor	Nilotinib	Autophagy	Inhibits autophagy negative regulator ABL1 and induces neuroprotective autophagy to reduce tau and improve astrocytic function	P301L Tau mice	Currently in AD Phase II clinical trial, NCT02947893	Hebron et al., 2018
Farnesyltrans ferase inhibitor	Lonafarnib	Autophagy	Farnesyltransferase inhibition prevents Rhes-mediated tau accumulation and activates autophagy	rTg4510, AD patient-derived human iPSC-differentiated neurons	Already in use in human patients for treating cancer	Hernandez et al., 2019
TFEB activators	Curcumin Analog C1	Activation of TFEB and autophagy-lysosomal pathways	Reduced the accumulation of phosphorylated tau	Mouse and rat primary neurons, 5xFAD, P301S, and 3xTg-AD mice	Multiple trials completed or currently in AD Phase II clinical trials	Song et al., 2020
TFEB activators	Flavonol Fisetin	Activation of TFEB, autophagy-lysosomal pathways	Induced activation of TFEB and autophagic degradation of phosphorylated tau	293T cells co-transfected with plasmids expressing tau and constitutively active HA-GSK-3β-S9A	Currently in AD Phase II clinical trial, NCT02380573	Currais et al., 2014
TFEB activators	Flubendazole	Activation of TFEB, autophagy-lysosomal pathways	Reduced the accumulation of phosphorylated tau	N2a cells	N/A	Chauhan et al., 2015
TFEB activators	Bromhexine	Activation of TFEB, autophagy-lysosomal pathways	Reduced the accumulation of phosphorylated tau	N2a cells	N/A	Chauhan et al., 2015
TFEB activators	Optogenetics	Activation of TFEB, autophagy-lysosomal pathways	Reduced the accumulation of phosphorylated tau	N2a cells, AD patient-derived human iPSC-differentiated neurons	N/A	Binder et al., 2020
Tau antibodies	Various antibodies	Lysosomal degradative pathways	Reduce oligomerization of tau and enhance tau degradation through AELN inside neurons or microglia	Various transgenic mice	Currently in Phase I or II AD clinical trials	Congdon and Sigurdsson, 2018

Phase II clinical trial of lithium has just ended in January 2020, and the results are published on ClinicalTrials.gov (NCT02129348). Many patients with AD did not show any response or showed a minimal response to lithium for agitation, aggression, or psychosis. Some of them had intolerable side effects, including the increased risk of mortality (60–70% higher than the placebo group), although the clinically used dose was 150–600 mg. A Brazilian group tested a micro-dose of lithium at 300 μg in AD patients for 15 months and found no decreased performance, thus recommending the application of micro-dose lithium in AD clinical trials.

Leuco-methylthioninium bis (hydromethanesulphonate) (LMTM), which is a stable reduced derivative of methylene blue, has gone through four Phase III trials by TauRx. In the first three trials (NCT01689246 for mild to moderate AD patients, NCT01689233 for mild AD patients, and NCT01626378 for subjects with behavioral variant frontotemporal dementia (bvFTD)), patients who took LMTM at 100 mg or 125 mg twice a day as their only therapy performed significantly better on cognitive and functional tests than those who took 8 mg, although with small group size and questionable subset analysis (Gauthier et al., 2016; Wilcock et al., 2018). Further analysis from NCT01689246, NCT01689233, and NCT01626378 show that the lowest dose (8 mg/day) of LMTM produced statistically significant concentration-dependent effects on the clinical decline and brain atrophy in both AD and bvFTD (Schelter et al., 2019; Shiells et al., 2020). In addition, TauRx recently finished a Phase III open-label extension trial (NCT02245568 in subjects with AD or bvFTD) for LMTM. TauRx is currently planning a new clinical trial, named LUCIDITY, where it uses FDG-PEG imaging and a composite cognitive/functional clinical psychometric scale to confirm the efficacy of LMTM as monotherapy at 8 and 16 mg/day. Another Phase II clinical trial (NCT02380573) for methylene blue in healthy aging, MCI, and AD was being conducted by Texas Alzheimer’s Research and Care Consortium. Unfortunately, this study has been suspended for now due to a funding issue.

Interestingly, VER-155008, a Hsc70 Chaperone inhibitor, did not inhibit PHF-tau degradation, rather increased reduction of PHF-tau, reversed axonal degeneration, and recovered memory function in 5xFAD mice, suggesting Hsc70 inhibition can lead to inhibition of UPS and CMA and shift to macroautophagy pathway. However, macroautophagy activation was not examined in that study (Yang and Tohda, 2018).

Another important aspect for enhancing tau removal is to promote tau degradation inside neurons or microglia using tau antibodies through active or passive immunotherapies [reviewed in Congdon and Sigurdsson (2018)]. It has been shown that anti-tau antibodies can enter neurons and co-localize with endosomes and lysosomes. Our group has recently reported that utilizing the Virus-like Particle (VLP) platform to generate vaccines targeting pT181 tau can induce a robust and long-lasting anti-pT181 antibody response in the sera and the brains of both non-transgenic and rTG4510 mouse model of FTD (Maphis et al., 2019). pT181-Qß vaccination reduced both soluble and insoluble pTau in the hippocampus and cortex, prevented hippocampal and corpus callosum atrophy, and rescued cognitive dysfunction in 4-month -old rTg4510 mice. This study proposes a VLP vaccine-based approach in the prevention and treatment of tauopathies. Although the exact mechanisms remain to be elucidated, it has been proposed that anti-tau antibodies can bind to extracellular tau and enter neurons through bulk endocytosis or receptor-mediated endocytosis and degrade tau through AELN (Congdon and Sigurdsson, 2018).

Microglia have also been shown to efficiently phagocytose and degrade AT8-positive human tau that is passively released from unfixed frozen brain sections of P301L transgenic mice and human AD brain sections and remove NFTs from P301S tauopathy mouse brain sections (Luo et al., 2015). Microglial phagocytosis of tau was enhanced by anti-tau monoclonal antibody MC1 – specific for conformational epitopes on PHF tau in an Fcγ-receptor-dependent manner. Another study also confirmed an anti-pS396-tau antibody C10.2-mediated clearance of tau in primary mouse microglial cultures needs Fcγ-receptor binding for antibody uptake and requires effector function and functional lysosome for internalized antibody-bound tau degradation (Andersson et al., 2019). Although another study argued that antibody effector function and microglia involvement is not required for antibody-mediated targeting of tau in vivo since only full-effector antibody promoted tau uptake into microglia and subsequently induced proinflammatory cytokine secretion, which can be detrimental to neurons, thus proposing effectorless tau antibodies might be safer for a therapeutic purpose (Lee et al., 2016).

### Preventing Tau Transmission Through AELN

Since neuron-to-neuron, neuron-to-glia tau transmission is an emerging field; there have not been many attempts yet to prevent tau transmission.

It has been shown that various antibodies targeting different regions of tau can exhibit a differential inhibitory effect on tau uptake and transmission between neurons. Antibodies targeting N-terminal (Tau13) and Mid-domain of tau (6C5 and HT7) can prevent internalization of tau, while antibody targeting C-terminal tau cannot. Phosphorylation-dependent (40E8 and p396) and C-terminal half (4E4) antibodies can also reduce tau internalization, although to a lesser extent. 6C5, the mid-domain targeting antibody, was able to block tau uptake and neuron-to-neuron propagation of tau in a microfluidic device and primary mouse neurons. It will be interesting to see if these antibodies have the same effects *in vivo* (Nobuhara et al., 2017).

Based on the current literature on AELN-mediated tau propagation, potential therapeutic interventions for preventing tau transmission can be summarized as follows (Table 6):

**TABLE 6:** Approaches to prevent tau transmission through AELN.

**Category**	**Name of interventions**	**Affected pathways**	**Mechanism of action**	**Models tested**	**References**
Tau acetylation inhibitor	p300 Inhibitor 37892	Degradative Autophagy, tau secretion (unknown pathway)	Inhibits p300/CBP, reduces tau acetylation, increases autophagy flux, and dampens tau secretion	Tau fibrils and AAV-Cre or AAV-GFP into 4–5-month-old PS19 mice carrying p300^F/F^ and CBP^F/F^	Chen et al., 2020
Tau antibodies	Various antibodies	Tau uptake and transmission	Prevent tau uptake and neuron-to-neuron transmission	Microfluidic chamber and primary mouse neurons	Nobuhara et al., 2017
nSMase2 inhibitor	Cambinol	Extracellular Vesicle/Exosome	Suppresses extracellular vesicle (EV) production while reducing tau seed propagation	N2a cells	Bilousova et al., 2018
HSPG inhibition	Heparin	HSPG-mediated tau secretion, uptake, and transmission	Reduced tau secretion from neurons and tau uptake by neurons and astrocytes	Primary mouse astrocytes	Martini-Stoica et al., 2018

(1) p300 inhibitor 37892, a small molecule inhibitor identified through screening, has been shown to inhibit p300/CBP, prevent tau acetylation, promote tau degradation through autophagy, and reduce tau secretion through an unidentified pathway.
(2) Cambinol, a nSMase2 inhibitor, was shown to suppress extracellular vesicle formation and tau secretion.
(3) Boosting astroglial TFEB activation since astroglial TFEB was involved in attenuating extracellular tau propagation and upregulating extracellular tau degradation.
(4) Inhibiting microglial BIN1 isoform 9 since it favors tau spreading.
(5) Inhibiting HSPG through Heparin, considering the involvement of HSPG in tau secretion, uptake, and transmission in neurons and astrocytes.

## Discussion

The degradation and propagation of tau through AELN is a complex process, and new studies are emerging to rapidly change the current state of knowledge.

Different isoforms and various post-translationally modified tau favor certain degradative pathways inside neurons, and different strains of tau also exhibit the distinct capacity of propagation in tauopathies. Considering the heterogenicity within tauopathies or even within the single patient, precision medicine needs to be taken into consideration when determining the therapeutic strategy for tauopathy patients.

On the other hand, autophagy plays a dual role in tau pathology: First, degrading pathological tau inside neurons and microglia; Second, mediating the unconventional secretion of phosphorylated tau, although autophagy-based unconventional secretion of tau needs further investigation both *in vitro* and *in vivo.*

In addition, neuronal and microglial BIN1 isoforms seem to have opposite functions. Neuronal BIN1 inhibits RAB5 activation and endosome formation to reduce tau propagation inside neurons, while microglial BIN1 isoform 9 is shown to promote tau propagation by incorporating tau into exosomes.

There are also multiple molecular pathways mediating tau release from neurons and tau internalization into neurons and glial cells. Therefore, investigating the molecular pathways mediating the degradation and transmission of the most neurotoxic form of tau by AELN will be beneficial to finding the novel therapeutic targets in AD and related tauopathies.

In this review, we attempted to list the potential targets and those that are already being examined in clinical trials, including tau-targeting antibodies. The literature on different modes of antibody-mediated clearance is a vast topic, and the goal of this review is not to go into the depth of any one type of mechanism in detail. Rather, we attempted to provide a generic overview of the current status of all the studies relevant to this topic and encourage readers to refer to other relevant reviews on specific topics.

## Author Contributions

SJ and KB wrote the manuscript. Both authors contributed to the article and approved the submitted version.

## Conflict of Interest

The authors declare that the research was conducted in the absence of any commercial or financial relationships that could be construed as a potential conflict of interest.

## References

[B1] Andersson C. R. Falsig J. Stavenhagen J. B. Christensen S. Kartberg F. Rosenqvist N. (2019). Antibody-mediated clearance of tau in primary mouse microglial cultures requires Fcγ-receptor binding and functional lysosomes. *Sci. Rep.* 9 1–12. 10.1038/s41598-019-41105-4 30874605 PMC6420568

[B2] Ando K. Brion J. P. Stygelbout V. Suain V. Authelet M. Dedecker R. (2013). Clathrin adaptor CALM/PICALM is associated with neurofibrillary tangles and is cleaved in Alzheimer’s brains. *Acta Neuropathol.* 125 861–878. 10.1007/s00401-013-1111-z 23589030

[B3] Ando K. Tomimura K. Sazdovitch V. Suain V. Yilmaz Z. Authelet M. (2016). Level of PICALM, a key component of clathrin-mediated endocytosis, is correlated with levels of phosphotau and autophagy-related proteins and is associated with tau inclusions in AD, PSP and Pick disease. *Neurobiol. Dis.* 94 32–43. 10.1016/j.nbd.2016.05.017 27260836

[B4] Asai H. Ikezu S. Tsunoda S. Medalla M. Luebke J. Haydar T. (2015). Depletion of microglia and inhibition of exosome synthesis halt tau propagation. *Nat. Neurosci.* 18 1584–1593. 10.1038/nn.4132 26436904 PMC4694577

[B5] Avila J. Gómez De Barreda E. Engel T. Lucas J. J. Hernández F. (2010). Tau phosphorylation in hippocampus results in toxic gain-of-function. *Biochem. Soc. Trans.* 38 977–980. 20658988 10.1042/BST0380977

[B6] Bi X. Haque T. S. Zhou J. Skillman A. G. Lin B. Lee C. E. (2002). Novel cathepsin D inhibitors block the formation of hyperphosphorylated tau fragments in hippocampus. *J. Neurochem.* 74 1469–1477. 10.1046/j.1471-4159.2000.0741469.x 10737603

[B7] Bilousova T. Elias C. Miyoshi E. Alam M. P. Zhu C. Campagna J. (2018). Suppression of tau propagation using an inhibitor that targets the DK-switch of nSMase2. *Biochem. Biophys. Res. Commun.* 499 751–757. 10.1016/j.bbrc.2018.03.209 29604274 PMC5956110

[B8] Binder J. L. Chander P. Deretic V. Weick J. P. Bhaskar K. (2020). Optical induction of autophagy via Transcription factor EB (TFEB) reduces pathological tau in neurons. *PLoS One* 15:e0230026. 10.1371/journal.pone.0230026 32208437 PMC7092971

[B9] Boland B. Yu W. H. Corti O. Mollereau B. Henriques A. Bezard E. (2018). Promoting the clearance of neurotoxic proteins in neurodegenerative disorders of ageing. *Nat. Rev. Drug Discov.* 17 660–688. 10.1038/nrd.2018.109 30116051 PMC6456907

[B10] Braak H. Braak E. (1995). Staging of alzheimer’s disease-related neurofibrillary changes. *Neurobiol. Aging* 16 271–278. 10.1016/0197-4580(95)00021-6 7566337

[B11] Caballero B. Wang Y. Diaz A. Tasset I. Juste Y. R. Stiller B. (2018). Interplay of pathogenic forms of human tau with different autophagic pathways. *Aging Cell* 17:e12692. 10.1111/acel.12692 29024336 PMC5770880

[B12] Calafate S. Flavin W. Verstreken P. Moechars D. (2016). Loss of bin1 promotes the propagation of tau pathology. *Cell Rep.* 17 931–940. 10.1016/j.celrep.2016.09.063 27760323

[B13] Chang S. H. Jung I. S. Han G. Y. Kim N. H. Kim H. J. Kim C. W. (2013). Proteomic profiling of brain cortex tissues in a Tau transgenic mouse model of Alzheimer’s disease. *Biochem. Biophys. Res. Commun.* 430 670–675. 10.1016/j.bbrc.2012.11.093 23211594

[B14] Chauhan S. Ahmed Z. Bradfute S. B. Arko-Mensah J. Mandell M. A. Won Choi S. (2015). Pharmaceutical screen identifies novel target processes for activation of autophagy with a broad translational potential. *Nat. Commun.* 6:8620. 10.1038/ncomms9620 26503418 PMC4624223

[B15] Chauhan S. Goodwin J. G. Chauhan S. Manyam G. Wang J. Kamat A. M. (2013). ZKSCAN3 is a master transcriptional repressor of autophagy. *Mol. Cell* 50 16–28. 10.1016/j.molcel.2013.01.024 23434374 PMC3628091

[B16] Chen X. Li Y. Wang C. Tang Y. Mok S.-A. Tsai R. M. (2020). Promoting tau secretion and propagation by hyperactive p300/CBP via autophagy-lysosomal pathway in tauopathy. *Mol. Neurodegen.* 15:2. 10.1186/s13024-019-0354-0 31906970 PMC6945522

[B17] Clavaguera F. Bolmont T. Crowther R. A. Abramowski D. Frank S. Probst A. (2009). Transmission and spreading of tauopathy in transgenic mouse brain. *Nat. Cell Biol.* 11 909–913. 10.1038/ncb1901 19503072 PMC2726961

[B18] Cocucci E. Meldolesi J. (2015). Ectosomes and exosomes: shedding the confusion between extracellular vesicles. *Trends Cell Biol.* 25 364–372. 10.1016/j.tcb.2015.01.004 25683921

[B19] Colacurcio D. J. Nixon R. A. (2016). Disorders of lysosomal acidification—The emerging role of v-ATPase in aging and neurodegenerative disease. *Ageing Res. Rev.* 32 75–88. 10.1016/j.arr.2016.05.004 27197071 PMC5112157

[B20] Congdon E. E. Sigurdsson E. M. (2018). Tau-targeting therapies for Alzheimer disease. *Nat. Rev. Neurol.* 14 399–415. 10.1038/s41582-018-0013-z 29895964 PMC6463489

[B21] Congdon E. E. Wu J. W. Myeku N. Figueroa Y. H. Herman M. Marinec P. S. (2012). Methylthioninium chloride (methylene blue) induces autophagy and attenuates tauopathy in vitro and in vivo. *Autophagy* 8 609–622. 10.4161/auto.19048 22361619 PMC3405840

[B22] Coppola G. Chinnathambi S. Lee J. J. Dombroski B. A. Baker M. C. Soto-Ortolaza A. I. (2012). Evidence for a role of the rare p.A152T variant in MAPT in increasing the risk for FTD-spectrum and Alzheimer’s diseases. *Hum. Mol. Genet.* 21 3500–3512. 10.1093/hmg/dds161 22556362 PMC3392107

[B23] Crotti A. Sait H. R. McAvoy K. M. Estrada K. Ergun A. Szak S. (2019). BIN1 favors the spreading of Tau via extracellular vesicles. *Sci. Rep.* 9 1–20. 10.1038/s41598-019-45676-0 31263146 PMC6603165

[B24] Cuervo A. M. Wong E. (2014). Chaperone-mediated autophagy: roles in disease and aging. *Cell Res.* 24 92–104. 10.1038/cr.2013.153 24281265 PMC3879702

[B25] Currais A. Prior M. Dargusch R. Armando A. Ehren J. Schubert D. (2014). Modulation of p25 and inflammatory pathways by fisetin maintains cognitive function in Alzheimer’s disease transgenic mice. *Aging Cell* 13 379–390. 10.1111/acel.12185 24341874 PMC3954948

[B26] Dall’Armi C. Hurtado-Lorenzo A. Tian H. Morel E. Nezu A. Chan R. B. (2010). The phospholipase D1 pathway modulates macroautophagy. *Nat. Commun.* 1 1–11. 10.1038/ncomms1144 21266992 PMC3328354

[B27] De Calignon A. Polydoro M. Suárez-Calvet M. William C. Adamowicz D. H. Kopeikina K. J. (2012). Propagation of tau pathology in a model of early Alzheimer’s disease. *Neuron* 73 685–697. 10.1016/j.neuron.2011.11.033 22365544 PMC3292759

[B28] Dice J. F. (1982). Altered degradation of proteins microinjected into senescent human fibroblasts. *J. Biol. Chem.* 257 14624–14627. 7174658

[B29] Dickson D. W. Baker M. Rademakers R. (2010). Common variant in GRN is a genetic risk factor for hippocampal sclerosis in the elderly. *Neurodegen. Dis.* 7 170–174. 10.1159/000289231 20197700 PMC2859236

[B30] Dikic I. (2017). Proteasomal and autophagic degradation systems. *Annu. Rev. Biochem.* 86 193–224. 10.1146/annurev-biochem-061516-044908 28460188

[B31] Dixit R. Ross J. L. Goldman Y. E. Holzbaur E. L. F. (2008). Differential regulation of dynein and kinesin motor proteins by tau. *Science* 319 1086–1089. 10.1126/science.1152993 18202255 PMC2866193

[B32] Dujardin S. Bégard S. Caillierez R. Lachaud C. Delattre L. Carrier S. (2014). Ectosomes: a new mechanism for non-exosomal secretion of tau protein. *PLoS One* 9:e100760. 10.1371/journal.pone.0100760 24971751 PMC4074092

[B33] Dupont N. Jiang S. Pilli M. Ornatowski W. Bhattacharya D. Deretic V. (2011). Autophagy-based unconventional secretory pathway for extracellular delivery of IL-1 beta. *EMBO J.* 30 4701–4711. 10.1038/emboj.2011.398 22068051 PMC3243609

[B34] Falcon B. Noad J. McMahon H. Randow F. Goedert M. (2018a). Galectin-8-mediated selective autophagy protects against seeded tau aggregation. *J. Biol. Chem.* 293 2438–2451. 10.1074/jbc.M117.809293 29282296 PMC5818177

[B35] Falcon B. Zhang W. Murzin A. G. Murshudov G. Garringer H. J. Vidal R. (2018b). Structures of filaments from Pick’s disease reveal a novel tau protein fold. *Nature* 561 137–140. 10.1038/s41586-018-0454-y 30158706 PMC6204212

[B36] Falcon B. Zivanov J. Zhang W. Murzin A. G. Garringer H. J. Vidal R. (2019). Novel tau filament fold in chronic traumatic encephalopathy encloses hydrophobic molecules. *Nature* 568 420–423. 10.1038/s41586-019-1026-5 30894745 PMC6472968

[B37] Farfel-Becker T. Roney J. C. Cheng X.-T. Li S. Cuddy S. R. Sheng Z.-H. (2019). Neuronal soma-derived degradative lysosomes are continuously delivered to distal axons to maintain local degradation capacity. *Cell Rep.* 28 51.e4–64.e4. 10.1016/j.celrep.2019.06.013 31269450 PMC6696943

[B38] Farg M. A. Sundaramoorthy V. Sultana J. M. Yang S. Atkinson R. A. K. Levina V. (2014). C9ORF72, implicated in amytrophic lateral sclerosis and frontotemporal dementia, regulates endosomal trafficking. *Hum. Mol. Genet.* 23 3579–3595. 10.1093/hmg/ddu068 24549040 PMC4049310

[B39] Ferrer I. Aguiló García M. Carmona M. Andrés-Benito P. Torrejón-Escribano B. Garcia-Esparcia P. (2019). Involvement of oligodendrocytes in tau seeding and spreading in tauopathies. *Front. Aging Neurosci.* 11:112. 10.3389/fnagi.2019.00112 31191295 PMC6546889

[B40] Fitzpatrick A. W. P. Falcon B. He S. Murzin A. G. Murshudov G. Garringer H. J. (2017). Cryo-EM structures of tau filaments from Alzheimer’s disease. *Nature* 547 185–190. 10.1038/nature23002 28678775 PMC5552202

[B41] Frost B. Jacks R. L. Diamond M. I. (2009). Propagation of Tau misfolding from the outside to the inside of a cell. *J. Biol. Chem.* 284 12845–12852. 10.1074/jbc.M808759200 19282288 PMC2676015

[B42] Fuster-Matanzo A. Hernández F. Ávila J. (2018). Tau spreading mechanisms; Implications for dysfunctional tauopathies. *Int. J. Mol. Sci.* 19:645. 10.3390/ijms19030645 29495325 PMC5877506

[B43] Gauthier S. Feldman H. H. Schneider L. S. Wilcock G. K. Frisoni G. B. Hardlund J. H. (2016). Efficacy and safety of tau-aggregation inhibitor therapy in patients with mild or moderate Alzheimer’s disease: a randomised, controlled, double-blind, parallel-arm, phase 3 trial. *Lancet* 388 2873–2884. 10.1016/S0140-6736(16)31275-2 27863809 PMC5164296

[B44] Gee H. Y. Noh S. H. Tang B. L. Kim K. H. Lee M. G. (2011). Rescue of delta F508-CFTR trafficking via a GRASP-dependent unconventional secretion pathway. *Cell* 146 746–760. 10.1016/j.cell.2011.07.021 21884936

[B45] Gibbons G. S. Lee V. M. Y. Trojanowski J. Q. (2019). Mechanisms of cell-to-cell transmission of pathological tau: a review. *JAMA Neurol.* 76 101–108. 10.1001/jamaneurol.2018.2505 30193298 PMC6382549

[B46] Götz J. Halliday G. Nisbet R. M. (2019). Molecular Pathogenesis of the Tauopathies. *Annu. Rev. Pathol. Mech. Dis.* 14 239–261. 10.1146/annurev-pathmechdis-012418-012936 30355155

[B47] Guillozet-Bongaarts A. L. Glajch K. E. Libson E. G. Cahill M. E. Bigio E. Berry R. W. (2007). Phosphorylation and cleavage of tau in non-AD tauopathies. *Acta Neuropathol.* 113 513–520. 10.1007/s00401-007-0209-6 17357802

[B48] Guo J. L. Lee V. M. Y. (2011). Seeding of normal tau by pathological tau conformers drives pathogenesis of Alzheimer-like tangles. *J. Biol. Chem.* 286 15317–15331. 10.1074/jbc.M110.209296 21372138 PMC3083182

[B49] Guo J. L. Narasimhan S. Changolkar L. He Z. Stieber A. Zhang B. (2016). Unique pathological tau conformers from alzheimer’s brains transmit tau pathology in nontransgenic mice. *J. Exp. Med.* 213 2635–2654. 10.1084/jem.20160833 27810929 PMC5110027

[B50] Hamano T. Gendron T. F. Causevic E. Yen S. H. Lin W. L. Isidoro C. (2008). Autophagic-lysosomal perturbation enhances tau aggregation in transfectants with induced wild-type tau expression. *Eur. J. Neurosci.* 27 1119–1130. 10.1111/j.1460-9568.2008.06084.x 18294209

[B51] Hebron M. L. Javidnia M. Moussa C. E. H. (2018). Tau clearance improves astrocytic function and brain glutamate-glutamine cycle. *J. Neurol. Sci.* 391 90–99. 10.1016/j.jns.2018.06.005 30103978

[B52] Hernandez I. Luna G. Rauch J. N. Reis S. A. Giroux M. Karch C. M. (2019). A farnesyltransferase inhibitor activates lysosomes and reduces tau pathology in mice with tauopathy. *Sci. Transl. Med.* 11:eaat3005. 10.1126/scitranslmed.aat3005 30918111 PMC7961212

[B53] Himmler A. Drechsel D. Kirschner M. W. Martin D. W. (1989). Tau consists of a set of proteins with repeated C-terminal microtubule-binding domains and variable N-terminal domains. *Mol. Cell. Biol.* 9 1381–1388. 10.1128/mcb.9.4.1381 2498649 PMC362554

[B54] Holler C. J. Davis P. R. Beckett T. L. Platt T. L. Webb R. L. Head E. (2014). Bridging integrator 1 (BIN1) protein expression increases in the alzheimer’s disease brain and correlates with neurofibrillary tangle pathology. *J. Alzheimers Dis.* 42 1221–1227. 10.3233/JAD-132450 25024306 PMC4198456

[B55] Holmes B. B. DeVos S. L. Kfoury N. Li M. Jacks R. Yanamandra K. (2013). Heparan sulfate proteoglycans mediate internalization and propagation of specific proteopathic seeds. *Proc. Natl. Acad. Sci. U.S.A.* 110 E3138–E3147. 10.1073/pnas.1301440110 23898162 PMC3746848

[B56] Hutton M. Lendon C. L. Rizzu P. Baker M. Froelich S. Houlden H. H. (1998). Association of missense and 5’-splice-site mutations in tau with the inherited dementia FTDP-17. *Nature* 393 702–704. 10.1038/31508 9641683

[B57] Iba M. Guo J. L. McBride J. D. Zhang B. Trojanowski J. Q. Lee V. M. Y. (2013). Synthetic tau fibrils mediate transmission of neurofibrillary tangles in a transgenic mouse model of alzheimer’s-like tauopathy. *J. Neurosci.* 33 1024–1037. 10.1523/JNEUROSCI.2642-12.2013 23325240 PMC3575082

[B58] Ikeda K. Akiyama H. Arai T. Kondo H. Haga C. Iritani S. (1998). Alz-50/Gallyas-positive lysosome-like intraneuronal granules in Alzheimer’s disease and control brains. *Neurosci. Lett.* 258 113–116. 10.1016/S0304-3940(98)00867-2 9875540

[B59] Ikeda K. Akiyama H. Arai T. Kondo H. Haga C. Tsuchiya K. (2000). Neurons containing Alz-50-immunoreactive granules around the cerebral infarction: evidence for the lysosomal degradation of altered tau in human brain? *Neurosci. Lett.* 284 187–189. 10.1016/S0304-3940(00)01009-0 10773430

[B60] Iqbal K. Liu F. Gong C.-X. (2016). *Tau and Neurodegenerative Disease: The Story So Far.* Berlin: Nature Publishing Group. 10.1038/nrneurol.2015.225 26635213

[B61] Jiang T. Yu J. T. Zhu X. C. Zhang Q. Q. Cao L. Wang H. F. (2014). Temsirolimus attenuates tauopathy in vitro and in vivo by targeting tau hyperphosphorylation and autophagic clearance. *Neuropharmacology* 85 121–130. 10.1016/j.neuropharm.2014.05.032 24880087

[B62] Johansen T. Lamark T. (2020). Selective autophagy: ATG8 family proteins, LIR motifs and cargo receptors. *J. Mol. Biol.* 432 80–103. 10.1016/j.jmb.2019.07.016 31310766

[B63] Kang S. Son S. M. Baik S. H. Yang J. Mook-Jung I. (2019). Autophagy-mediated secretory pathway is responsible for both normal and pathological tau in neurons. *J. Alzheimers Dis. JAD* 70 667–680. 10.3233/JAD-190180 31256134

[B64] Katsinelos T. Zeitler M. Dimou E. Karakatsani A. Müller H. M. Nachman E. (2018). Unconventional secretion mediates the trans-cellular spreading of tau. *Cell Rep.* 23 2039–2055. 10.1016/j.celrep.2018.04.056 29768203

[B65] Katsumata K. Nishiyama J. Inoue T. Mizushima N. Takeda J. Yuzaki M. (2010). Dynein-and activity-dependent retrograde transport of autophagosomes in neuronal axons. *Autophagy* 6 378–385. 10.4161/auto.6.3.11262 20150763

[B66] Kaushik S. Cuervo A. M. (2018). The coming of age of chaperone-mediated autophagy. *Nat. Rev. Mol. Cell Biol.* 19 365–381. 10.1038/s41580-018-0001-6 29626215 PMC6399518

[B67] Kraus A. Saijo E. Metrick M. A. Newell K. Sigurdson C. J. Zanusso G. (2019). Seeding selectivity and ultrasensitive detection of tau aggregate conformers of Alzheimer disease. *Acta Neuropathol.* 137 585–598. 10.1007/s00401-018-1947-3 30570675 PMC6426988

[B68] Krüger U. Wang Y. Kumar S. Mandelkow E.-M. (2012). Autophagic degradation of tau in primary neurons and its enhancement by trehalose. *Neurobiol. Aging* 33 2291–2305. 10.1016/J.NEUROBIOLAGING.2011.11.009 22169203

[B69] Lee H. R. Shin H. K. Park S. Y. Kim H. Y. Lee W. S. Rhim B. Y. (2014). Attenuation of β-amyloid-induced tauopathy via activation of CK2α/SIRT1: targeting for cilostazol. *J. Neurosci. Res.* 92 206–217. 10.1002/jnr.23310 24254769

[B70] Lee M. J. Lee J. H. Rubinsztein D. C. (2013). Tau degradation: the ubiquitin-proteasome system versus the autophagy-lysosome system. *Prog. Neurobiol.* 105 49–59. 10.1016/j.pneurobio.2013.03.001 23528736

[B71] Lee S. H. Le Pichon C. E. Adolfsson O. Gafner V. Pihlgren M. Lin H. (2016). Antibody-mediated targeting of tau in vivo does not require effector function and microglial engagement. *Cell Rep.* 16 1690–1700. 10.1016/j.celrep.2016.06.099 27475227

[B72] Lee V. M.-Y. Goedert M. Trojanowski J. Q. (2001). Neurodegenerative tauopathies. *Annu. Rev. Neurosci.* 24 1121–1159. 10.1146/annurev.neuro.24.1.1121 11520930

[B73] Li C. Götz J. (2017). Tau-based therapies in neurodegeneration: opportunities and challenges. *Nat. Rev. Drug Discov.* 16 863–883. 10.1038/nrd.2017.155 28983098

[B74] Liu L. Drouet V. Wu J. W. Witter M. P. Small S. A. Clelland C. (2012). Trans-synaptic spread of tau pathology in vivo. *PLoS One* 7:e0031302. 10.1371/journal.pone.0031302 22312444 PMC3270029

[B75] Luo W. Liu W. Hu X. Hanna M. Caravaca A. Paul S. M. (2015). Microglial internalization and degradation of pathological tau is enhanced by an anti-tau monoclonal antibody. *Sci. Rep.* 5:11161. 10.1038/srep11161 26057852 PMC4460904

[B76] Maphis N. Xu G. Kokiko-Cochran O. N. Jiang S. Cardona A. Ransohoff R. M. (2015). Reactive microglia drive tau pathology and contribute to the spreading of pathological tau in the brain. *Brain J. Neurol.* 138 (Pt 6), 1738–1755. 10.1093/brain/awv081 25833819 PMC4542622

[B77] Maphis N. M. Peabody J. Crossey E. Jiang S. Jamaleddin Ahmad F. A. Alvarez M. (2019). Qß Virus-like particle-based vaccine induces robust immunity and protects against tauopathy. *NPJ Vaccines* 4:26. 10.1038/s41541-019-0118-4 31231552 PMC6547647

[B78] Martini-Stoica H. Cole A. L. Swartzlander D. B. Chen F. Wan Y. W. Bajaj L. (2018). TFEB enhances astroglial uptake of extracellular tau species and reduces tau spreading. *J. Exp. Med.* 215 2355–2377. 10.1084/jem.20172158 30108137 PMC6122971

[B79] Menzies F. M. Fleming A. Caricasole A. Bento C. F. Andrews S. P. Ashkenazi A. (2017). Autophagy and neurodegeneration: pathogenic mechanisms and therapeutic opportunities. *Neuron* 93 1015–1034. 10.1016/j.neuron.2017.01.022 28279350

[B80] Merezhko M. Brunello C. A. Yan X. Vihinen H. Jokitalo E. Uronen R. L. (2018). Secretion of tau via an unconventional non-vesicular mechanism. *Cell Rep.* 25 2027.e4–2035.e4. 10.1016/j.celrep.2018.10.078 30463001

[B81] Min S. W. Cho S. H. Zhou Y. Schroeder S. Haroutunian V. Seeley W. W. (2010). Acetylation of tau inhibits its degradation and contributes to tauopathy. *Neuron* 67 953–966. 10.1016/j.neuron.2010.08.044 20869593 PMC3035103

[B82] Moreau K. Fleming A. Imarisio S. Lopez Ramirez A. Mercer J. L. Jimenez-Sanchez M. (2014). PICALM modulates autophagy activity and tau accumulation. *Nat. Commun.* 5:4998. 10.1038/ncomms5998 25241929 PMC4199285

[B83] Narasimhan S. Guo J. L. Changolkar L. Stieber A. McBride J. D. Silva L. V. (2017). Pathological tau strains from human brains recapitulate the diversity of tauopathies in nontransgenic mouse brain. *J. Neurosci.* 37 11406–11423. 10.1523/JNEUROSCI.1230-17.2017 29054878 PMC5700423

[B84] Nixon R. A. (2013). The role of autophagy in neurodegenerative disease. *Nat. Med.* 19 983–997. 10.1038/nm.3232 23921753

[B85] Nixon R. A. Wegiel J. Kumar A. Yu W. H. Peterhoff C. Cataldo A. (2005). Extensive involvement of autophagy in alzheimer disease: an immuno-electron microscopy study. *J. Neuropathol. Exp. Neurol.* 64 113–122. 10.1093/JNEN/64.2.113 15751225

[B86] Nobuhara C. K. DeVos S. L. Commins C. Wegmann S. Moore B. D. Roe A. D. (2017). Tau antibody targeting pathological species blocks neuronal uptake and interneuron propagation of tau in vitro. *Am. J. Pathol.* 187 1399–1412. 10.1016/j.ajpath.2017.01.022 28408124 PMC5455060

[B87] Paushter D. H. Du H. Feng T. Hu F. (2018). The lysosomal function of progranulin, a guardian against neurodegeneration. *Acta Neuropathol.* 136:1. 10.1007/s00401-018-1861-8 29744576 PMC6117207

[B88] Perea J. R. López E. Díez-Ballesteros J. C. Ávila J. Hernández F. Bolós M. (2019). Extracellular monomeric tau is internalized by astrocytes. *Front. Neurosci.* 13:442. 10.3389/fnins.2019.00442 31118883 PMC6504834

[B89] Perez S. E. He B. Nadeem M. Wuu J. Ginsberg S. D. Ikonomovic M. D. (2015). Hippocampal endosomal, lysosomal, and autophagic dysregulation in mild cognitive impairment: correlation with Aβ and tau pathology. *J. Neuropathol. Exp. Neurol.* 74 345–358. 10.1097/NEN.0000000000000179 25756588 PMC4366294

[B90] Perry D. C. Lehmann M. Yokoyama J. S. Karydas A. Lee J. J. Y. Coppola G. (2013). Progranulin mutations as risk factors for alzheimer disease. *JAMA Neurol.* 70 774–778. 10.1001/2013.jamaneurol.393 23609919 PMC3743672

[B91] Piras A. Collin L. Grüninger F. Graff C. Rönnbäck A. (2016). Autophagic and lysosomal defects in human tauopathies: analysis of post-mortem brain from patients with familial Alzheimer disease, corticobasal degeneration and progressive supranuclear palsy. *Acta Neuropathol. Commun.* 4:22. 10.1186/s40478-016-0292-9 26936765 PMC4774096

[B92] Polito V. A. Li H. Martini-Stoica H. Wang B. Yang L. Xu Y. (2014). Selective clearance of aberrant tau proteins and rescue of neurotoxicity by transcription factor EB. *EMBO Mol. Med.* 6, 1142–1160. 10.15252/emmm.201303671 25069841 PMC4197862

[B93] Pooler A. M. Phillips E. C. Lau D. H. W. Noble W. Hanger D. P. (2013). Physiological release of endogenous tau is stimulated by neuronal activity. *EMBO Rep.* 14 389–394. 10.1038/embor.2013.15 23412472 PMC3615658

[B94] Puangmalai N. Bhatt N. Montalbano M. Sengupta U. Gaikwad S. Ventura F. (2020). Internalization mechanisms of brain-derived tau oligomers from patients with Alzheimer’s disease, progressive supranuclear palsy and dementia with Lewy bodies. *Cell Death Dis.* 11:314. 10.1038/s41419-020-2503-3 32366836 PMC7198578

[B95] Rapoport M. Dawson H. N. Binder L. I. Vitek M. P. Ferreira A. (2002). Tau is essential to β-amyloid-induced neurotoxicity. *Proc. Natl. Acad. Sci. U.S.A.* 99 6364–6369. 10.1073/pnas.092136199 11959919 PMC122954

[B96] Rodríguez-Navarro J. A. Rodríguez L. Casarejos M. J. Solano R. M. Gómez A. Perucho J. (2010). Trehalose ameliorates dopaminergic and tau pathology in parkin deleted/tau overexpressing mice through autophagy activation. *Neurobiol. Dis.* 39 423–438. 10.1016/j.nbd.2010.05.014 20546895

[B97] Sahu R. Kaushik S. Clement C. C. Cannizzo E. S. Scharf B. Follenzi A. (2011). Microautophagy of cytosolic proteins by late endosomes. *Dev. Cell* 20 131–139. 10.1016/j.devcel.2010.12.003 21238931 PMC3025279

[B98] Saman S. Kim W. Raya M. Visnick Y. Miro S. Jackson B. (2011). Exosome-associated tau is secreted in tauopathy models and is selectively phosphorylated in cerebrospinal fluid in early alzheimer disease. *J. Biol. Chem.* 287 3842–3849. 10.1074/jbc.M111.277061 22057275 PMC3281682

[B99] Sanders D. W. Kaufman S. K. DeVos S. L. Sharma A. M. Mirbaha H. Li A. (2014). Distinct tau prion strains propagate in cells and mice and define different tauopathies. *Neuron* 82 1271–1288. 10.1016/j.neuron.2014.04.047 24857020 PMC4171396

[B100] Sardiello M. Palmieri M. Ronza A. Di Medina D. L. Valenza M. Gennarino V. A. (2009). A gene network regulating lysosomal biogenesis and function. *Science* 325 473–477. 10.1126/science.1174447 19556463

[B101] Schaeffer V. Goedert M. (2012). Stimulation of autophagy is neuroprotective in a mouse model of human tauopathy. *Autophagy* 8 1686–1687. 10.4161/auto.21488 22874558 PMC3494601

[B102] Schelter B. O. Shiells H. Baddeley T. C. Rubino C. M. Ganesan H. Hammel J. (2019). Concentration-dependent activity of hydromethylthionine on cognitive decline and brain atrophy in mild to moderate Alzheimer’s disease. *J. Alzheimers Dis.* 72 931–946. 10.3233/JAD-190772 31658058 PMC6918900

[B103] Schöll M. Lockhart S. N. Schonhaut D. R. O’Neil J. P. Janabi M. Ossenkoppele R. (2016). PET imaging of tau deposition in the aging human brain. *Neuron* 89 971–982. 10.1016/j.neuron.2016.01.028 26938442 PMC4779187

[B104] Settembre C. Di Malta C. Polito V. A. Arencibia M. G. Vetrini F. Erdin S. (2011). TFEB links autophagy to lysosomal biogenesis. *Science* 332 1429–1433. 10.1126/science.1204592 21617040 PMC3638014

[B105] Shiells H. Schelter B. O. Bentham P. Baddeley T. C. Rubino C. M. Ganesan H. (2020). Concentration-dependent activity of hydromethylthionine on clinical decline and brain atrophy in a randomized controlled trial in behavioral variant frontotemporal dementia. *J. Alzheimers Dis. JAD* 75 501–519. 10.3233/JAD-191173 32280089 PMC7306898

[B106] Shimada K. Motoi Y. Ishiguro K. Kambe T. Matsumoto S. E. Itaya M. (2012). Long-term oral lithium treatment attenuates motor disturbance in tauopathy model mice: implications of autophagy promotion. *Neurobiol. Dis.* 46 101–108. 10.1016/j.nbd.2011.12.050 22249108

[B107] Skibinski G. Parkinson N. J. Brown J. M. Chakrabarti L. Lloyd S. L. Hummerich H. (2005). Mutations in the endosomal ESCRTIII-complex subunit CHMP2B in frontotemporal dementia. *Nat. Genet.* 37 806–808. 10.1038/ng1609 16041373

[B108] Song J. Malampati S. Zeng Y. Durairajan S. S. K. Yang C. Tong B. C. (2020). A small molecule transcription factor EB activator ameliorates beta-amyloid precursor protein and Tau pathology in Alzheimer’s disease models. *Aging Cell* 19:e13069. 10.1111/acel.13069 31858697 PMC6996953

[B109] Tardivel M. Bégard S. Bousset L. Dujardin S. Coens A. Melki R. (2016). Tunneling nanotube (TNT)-mediated neuron-to neuron transfer of pathological Tau protein assemblies. *Acta Neuropathol. Commun.* 4:117. 10.1186/s40478-016-0386-4 27809932 PMC5096005

[B110] Thorburn J. Horita H. Redzic J. Hansen K. Frankel A. E. Thorburn A. (2009). Autophagy regulates selective HMGB1 release in tumor cells that are destined to die. *Cell Death Diff.* 16 175–183. 10.1038/cdd.2008.143 18846108 PMC2605182

[B111] Trojanowski J. Q. Lee V. M. Y. (2005). Pathological tau: a loss of normal function or a gain in toxicity? *Nat. Neurosci.* 8 1136–1137. 10.1038/nn0905-1136 16127446

[B112] Uemura N. Uemura M. T. Luk K. C. Lee V. M. Y. Trojanowski J. Q. (2020). Cell-to-cell transmission of tau and α-synuclein. *Trends Mol. Med.* 26 936–952. 10.1016/j.molmed.2020.03.012 32371172 PMC7529725

[B113] Uytterhoeven V. Deaulmerie L. Verstreken P. (2018). Increased endosomal microautophagy reduces Tau driven synaptic dysfunction. *Front. Neurosci.* 12:102 10.3389/conf.fnins.2018.95.00102

[B114] Uytterhoeven V. Lauwers E. Maes I. Miskiewicz K. Melo M. N. Swerts J. (2015). Hsc70-4 deforms membranes to promote synaptic protein turnover by endosomal microautophagy. *Neuron* 88 735–748. 10.1016/j.neuron.2015.10.012 26590345

[B115] Van Acker Z. P. Bretou M. Annaert W. (2019). Endo-lysosomal dysregulations and late-onset Alzheimer’s disease: impact of genetic risk factors. *Mol. Neurodegen.* 14:20. 10.1186/s13024-019-0323-7 31159836 PMC6547588

[B116] Vaz-Silva J. Gomes P. Jin Q. Zhu M. Zhuravleva V. Quintremil S. (2018). Endolysosomal degradation of Tau and its role in glucocorticoid-driven hippocampal malfunction. *EMBO J.* 37:e99084. 10.15252/embj.201899084 30166454 PMC6187216

[B117] Wang H. Wang R. Carrera I. Xu S. Lakshmana M. K. (2016). TFEB overexpression in the P301S model of tauopathy mitigates increased PHF1 levels and lipofuscin puncta and rescues memory deficits. *eNeuro* 3 9340–9351. 10.1523/ENEURO.0042-16.2016 27257626 PMC4876487

[B118] Wang Y. Balaji V. Kaniyappan S. Krüger L. Irsen S. Tepper K. (2017). The release and trans-synaptic transmission of Tau via exosomes. *Mol. Neurodegen.* 12:5. 10.1186/s13024-016-0143-y 28086931 PMC5237256

[B119] Wang Y. Mandelkow E. (2012). Degradation of tau protein by autophagy and proteasomal pathways. *Biochem. Soc. Trans.* 40 644–652. 10.1042/BST20120071 22817709

[B120] Wang Y. Martinez-Vicente M. Krüger U. Kaushik S. Wong E. Mandelkow E.-M. (2009). Tau fragmentation, aggregation and clearance: the dual role of lysosomal processing. *Hum. Mol. Genet.* 18 4153–4170. 10.1093/hmg/ddp367 19654187 PMC2758146

[B121] Wilcock G. K. Gauthier S. Frisoni G. B. Jia J. Hardlund J. H. Moebius H. J. (2018). Potential of low dose leuco-methylthioninium Bis(Hydromethanesulphonate) (LMTM) monotherapy for treatment of mild Alzheimer’s disease: cohort analysis as modified primary outcome in a phase III clinical trial. *J. Alzheimers Dis.* 61 435–457. 10.3233/JAD-170560 29154277 PMC5734125

[B122] World Alzheimer Report (2019). *World Alzheimer Report 2019: Attitudes to dementia; World Alzheimer Report 2019: Attitudes to Dementia.* Available at: www.daviddesigns.co.uk (accessed July 21, 2020).

[B123] Wu J. W. Herman M. Liu L. Simoes S. Acker C. M. Figueroa H. (2013). Small misfolded tau species are internalized via bulk endocytosis and anterogradely and retrogradely transported in neurons. *J. Biol. Chem.* 288 1856–1870. 10.1074/jbc.M112.394528 23188818 PMC3548495

[B124] Wu J. W. Hussaini S. A. Bastille I. M. Rodriguez G. A. Mrejeru A. Rilett K. (2016). Neuronal activity enhances tau propagation and tau pathology in vivo. *Nat. Neurosci.* 19 1085–1092. 10.1038/nn.4328 27322420 PMC4961585

[B125] Yamada K. Holth J. K. Liao F. Stewart F. R. Mahan T. E. Jiang H. (2014). Neuronal activity regulates extracellular tau in vivo. *J. Exp. Med.* 211 387–393. 10.1084/jem.20131685 24534188 PMC3949564

[B126] Yang X. Tohda C. (2018). Heat shock cognate 70 inhibitor, VER-155008, reduces memory deficits and axonal degeneration in a mouse model of Alzheimer’s disease. *Front. Pharmacol.* 9:48. 10.3389/fphar.2018.00048 29441022 PMC5797615

[B127] Yuan A. Kumar A. Peterhoff C. Duff K. Nixon R. A. (2008). Axonal transport rates in vivo are unaffected by tau deletion or overexpression in mice. *J. Neurosci.* 28 1682–1687. 10.1523/JNEUROSCI.5242-07.2008 18272688 PMC2814454

[B128] Zhang W. Falcon B. Murzin A. G. Fan J. Crowther R. A. Goedert M. (2019). Heparin-induced tau filaments are polymorphic and differ from those in alzheimer’s and pick’s diseases. *eLife* 8:e43584. 10.7554/eLife.43584 30720432 PMC6375701

[B129] Zhang W. Tarutani A. Newell K. L. Murzin A. G. Matsubara T. Falcon B. (2020). Novel tau filament fold in corticobasal degeneration. *Nature* 580 283–287. 10.1038/s41586-020-2043-0 32050258 PMC7148158

